# Supplementary material for: Assessment of sewer connectivity in the United States and its implications for equity in wastewater-based epidemiology
Source: PLOS Glob Public Health. 2024 Apr 17;4(4):e0003039. doi: 10.1371/journal.pgph.0003039 (PMC11023481; doi:10.1371/journal.pgph.0003039)
Supplement: S1 Appendix — (DOCX) [file pgph.0003039.s001.docx]

**S1 Appendix. Supplementary methods.**

**Dataset descriptions and data cleaning**

U.S. Census Bureau American Housing Survey 2019 and 2021

The American Housing Survey (AHS) National and Metropolitan Public Use Files were downloaded from Ref. [1–3]. The sample design, weighting, and error estimation survey is described in Refs. [4–6]. Briefly, the AHS is a biannual survey that assesses housing characteristics. Two samples of households are chosen every survey cycle, the Integrated National Sample and the Independent Metropolitan Sample. The Integrated National Sample consists of nationally representative households and includes an oversample of each of 15 largest metropolitan areas. The Independent Metropolitan Sample consists of representative households in 20 large metropolitan areas that are representative of the next largest 50 metropolitan areas. Ten of the 20 large metropolitan areas are sampled every other survey cycle (10 metropolitan areas surveyed every 2 years). The Integrated National Sample in 2019 included 86,257 selected representative housing units, of which about 63,500 met the AHS definition of a housing unit and were able to be interviewed, and in 2021 included 95,295 selected representative housing units, of which about 64,100 were interviewed. The Integrated Metropolitan Samples in 2019 and 2021 included around 3000 selected representative housing units in each metropolitan area, of which around 1000-3000 were eligible and were able to be interviewed (same criteria as in the Integrated National Sample).

Geographic data of each household available in the Public Use File included the census division; core based statistical area (CBSA) for the 15 largest metropolitan areas and the 20 large metropolitan areas in the metropolitan data. All other households are classified as in “all other metropolitan areas” or “not in a metropolitan area” (all other locations).

The race, ethnicity, and age of the householder (the person or one of the persons who is an owner or renter of the unit) was used to categorize the race, ethnicity, and age of the household. Due to the low numbers of households where the householder responded as having more than one race, we focused on households where householders identified as having a single race.

U.S. Census Bureau American Housing Survey National Sample 2013

The American Housing Survey National (2013) Use File was downloaded from Ref. [7]. The sample design, weighting, and error estimation survey is described in Ref [8]. The National Sample included 84,400 selected housing units representative nationally and including a supplemental sample of 15,553 housing units in the Chicago, Detroit, New York City, Northern New Jersey, and Philadelphia metropolitan statistical areas, of which about 71,600 met the definition of a housing unit and were able to be interviewed.

Geographic data of each household available in the Public Use File included the census division; census region; central city, urban, or rural status; and select metropolitan area codes. All other housing units are classified into “not in a metropolitan area” or had their code suppressed for confidentiality [9].

Florida Department of Health onsite sewage treatment and disposal system inspections

The locations of onsite sewage treatment and disposal systems inspected by Florida Department of Health reported June 2012 was downloaded from Ref. [10]. As of 2022, an inspection permit is required for construction [11] and inspection is recommended every 3 to 5 years after [12]. The number of systems inspected that are active at the latest inspection (after removing repeat inspections) is 601,430. The years of inspection are primarily 1998-2011, with some outliers as early as 1900, which may be old septic systems or typos, and as late as 2200, which are likely typos.

Utah Municipal Wastewater Planning Program (MWPP) Survey 2021

Data from the Utah Municipal Wastewater Planning Survey was provided by the Utah Department of Environment Quality, Division of Water Quality. The dataset includes an estimate of the population receiving collection by each utility that owns or operates a sanitary sewerage system and its latitude and longitude. The data gathered from treatment plant operators and participation is reported as mandatory [13], although only 71% of contacts responded [14]. We filtered the data to include any facility that has collection (this included collection only, treatment and collection, or small lagoons which are often collection and treatment). Manual correction of facility longitude and latitude is described in the supplementary methods.

Manual editing of facility longitude and latitude was done for 5 facilities’ that did not fall within the state of Utah when combining with the 2021 TIGER shapefile (BRIAN HEAD TOWN, CANYONG LAND IMPROVEMENT DISTRICT, HILL AFB (AMERICAN WATER), JORDANELLE SSD, PLAIN CITY) and 1 facility that did not report a latitude and longitude (PANGUITCH LAKE S. S. D.). For these facilities, the latitude and longitude were manually edited to be that of the location found in a Google Maps search of the facility name. If the Google Maps search found a city, then an arbitrarily chosen location in that city was used to obtain a latitude and longitude.

Minnesota Wastewater Infrastructure Needs Survey (WINS) 2021

Data for the Minnesota Wastewater Infrastructure Needs Survey [15] was provided by the Minnesota Pollution Control Agency. Communities (places and townships) responded to the question “Does your community have a collection system?” with “yes”, “no”, or no response. The survey did not receive a response from every community in Minnesota.

The Census Designated Place (CDP) name was obtained from the reported community name with manual changes reported in **S5 Table**.

Minnesota Subsurface Sewage Treatment Systems 2017

The Minnesota Subsurface Sewage Treatment Systems (SSTS) dataset reported the number of SSTS by local government units with a known SSTS program. Data was taken from Ref. [16].

Two hundred and eleven out of 2018 known SSTS programs submitted an annual report. Not all SSTS programs were able to be identified and contacts were not always provided. Fourteen reported having zero SSTS within their jurisdiction despite permitting SSTS in 2017. Data were reported to sometimes be estimates.

U.S. Environmental Protection Agency Clean Watersheds Needs Survey 2012

Data for the most recent Environmental Protection Agency (EPA) Clean Watersheds Needs Survey (CWNS) survey (2012) [17] was downloaded from [18] and extracted from a Microsoft Access database file to csv files using the mdbtools package (<https://github.com/mdbtools/mdbtools>). The survey is administered by the EPA and the States to assess funding needs for unfunded capital costs of treatment works projects that (1) address a water quality or related public health problem existing as of January 1, 2012, or is expected to occur within the next 20 years and (2) meet the CWNS documentation criteria. CWNS documentation criteria includes (1) description and location of the problem, (2) site-specific solution to the problem, and (3) detailed cost information for implementing the solution. Data is collected about (1) publicly owned wastewater collection and treatment facilities, (2) combined sewer overflow control facilities, (3) stormwater management activities, and (4) decentralized wastewater treatment facilities. Data is collected on (a) estimated needs, costs and technical information, (b) facility location and contact information, and (c) facility population served, flow, effluent, and unit process information. Supporting documentation was required for the entered data with some exceptions (for instance small communities could use a more simple form), and quality control was performed by reviewers to compare documentation with data entered in the system to ensure consistency of technical and cost data [19]. Data entry occurred from January to December 2012.

There was large variability in the level of effort and resources that each state put into the survey. New York, California, Florida, New Jersey, Maryland, Iowa, Minnesota, and Michigan likely invested heavily in participating in the survey or already had similar state-level data collection systems in place for getting comprehensive responses [10,15,16].

Facilities not included in the dataset are those in South Carolina, the Northern Mariana Islands, and American Samoa as they did not participate in the survey; facilities whose projects did not have documented solutions or cost estimates; privately owned wastewater facilities that serve privately owned industrial facilities, military installations, national parks, or other federal facilities; facilities on tribal lands and in Alaskan Native Villages, which were separately surveyed by the Indian Health Service; and facilities with projects that had existing funding prior to January 1, 2012. Additionally, larger facilities were more likely to be captured by the survey due to the resource requirements for obtaining the right documentation. To address this bias, the survey had decreased documentation needs for small communities (population <=10,000); despite this, the small community facilities were still underrepresented (pg. 2 of Ref. [17]).

We filtered the facilities to the current facilities of type “collection: combined sewers” or “collection: separate sewers”. The population receiving collection is taken from the reported total number of residents who are connected to a sewer system which empties into a treatment plant. This does not include non-resident populations, populations served by acceptable decentralized wastewater treatment systems, or populations connected to sewers that do not discharge to a treatment plant.

U.S. Census Bureau Island Areas Decennial Survey 2020

The Island Areas Decennial Survey surveys housing, social, and economic information for all housing units in Guam, the Northern Mariana Islands, the Virgin Islands, and American Samoa [20]. Data of general demographic characteristics (DP01), selected economic characteristics (DP03), and selected housing characteristics (DP04) were downloaded from <https://data.census.gov/>. Housing characteristics included data for both occupied and vacant housing units.

U.S. Census Bureau American Community Survey

The American Community Survey (ACS) is a monthly survey of a representative subsample of addresses in the US [21] on characteristics of populations and households. The ACS 5 year estimates (average over 5 years, less subject to fluctuations) for selected social characteristics (DP02), selected economic characteristics (DP03), and demographic and housing estimates (DP05) stratified county or county subdivision for select years were downloaded from <https://data.census.gov/>.

**Data analysis and visualization**

Florida Department of Health onsite sewage treatment and disposal system inspections dataset analysis

The number of septic tanks in each county subdivision was calculated as the number of septic permits whose latitude and longitude occurred inside the boundaries of each county subdivision according to the 2012 U.S. Census Bureau county subdivision TIGER shapefile. To calculate the fraction of a county subdivision connected to septic tanks, the number of septic tanks in each county subdivision was divided by the number of households in the county subdivision as reported by the 2012 American Community Survey 5-year estimate. Only county subdivisions with at least 5 households and 20 population size were used.

Utah Municipal Wastewater Planning Program Survey analysis

The population in each county receiving collection was calculated by summing the population receiving collection across all reported facilities in that county. The population in each county subdivision receiving collection was calculated by summing the population receiving collection across all reported facilities whose latitude and longitude fell within that county subdivision’s boundaries from the 2021 TIGER shapefile. To calculate the fraction of a county or county subdivision receiving sewer collection, the population receiving collection was divided by the population size of each county or county subdivision reported by the 2021 American Community Survey 5-year estimate. Only county subdivisions with at least 5 households and 20 population size were used.

U.S. Environmental Protection Agency Clean Watersheds Needs Survey analysis

To compare the EPA CWNS and AHS data in Core Based Statistical Areas (CBSA), counties were categorized into CBSAs using the February 2013 U.S. Census Bureau file. To calculate the fraction of a county receiving collection, the population size receiving collection in that county was divided by the population size of each county reported by the 2012 American Community Survey 5-year estimate. We flagged counties that had no facilities reported, which could either be because there were no facilities in that county or because of a lack of reporting, and counties that had >100% sewer connectivity by population (potentially due to misreporting or collection occurring across multiple counties) were set to 100% connectivity.

U.S. Census Bureau Island Areas Decennial Survey analysis

To calculate the fraction of a Census Designated Place (CDP) connected to sewers, the number of households connected to sewers was divided by the total number of households in a CDP. Only CDPs with at least 5 households and 20 population size were used.

**Generation of maps**

TIGER shapefiles from the U.S. Census Bureau in the closest matching year to the dataset were used for generating maps. Maps were generated using the geopandas package (version 0.12.2) in Python (version 3.11.0) and were displayed in the Mercator projection.

**Simulations of interacting populations and wastewater sampling**

Two interacting populations are modeled in a compartmental model with susceptible, infected, and recovered (SIR) compartments for each population. The number of susceptible, infected, and recovered individuals in population A (*S_A_*, *I_A_*, and *R_A_*, respectively) and in population B (*S_B_*, *I_B_*, and *R_B_*, respectively) are described by:

$$\frac{{dS}_{A}}{dt}=-\beta_{A}\left( 1-\varepsilon\right)I_{A}\frac{S_{A}}{N_{A}}-\beta_{B}\varepsilon I_{B}\frac{S_{A}}{N_{A}}$$

$$\frac{{dS}_{B}}{dt}=-\beta_{B}\left( 1-\varepsilon\right)I_{B}\frac{S_{B}}{N_{B}}-\beta_{A}\varepsilon I_{A}\frac{S_{B}}{N_{B}}$$

$$\frac{{dI}_{A}}{dt}=\beta_{A}\left( 1-\varepsilon\right)I_{A}\frac{S_{A}}{N_{A}}+\beta_{B}\varepsilon I_{B}\frac{S_{A}}{N_{A}}-\gamma_{I}I_{A}$$

$$\frac{{dI}_{B}}{dt}=\beta_{B}\left( 1-\varepsilon\right)I_{B}\frac{S_{B}}{N_{B}}+\beta_{A}\varepsilon I_{A}\frac{S_{B}}{N_{B}}-\gamma_{I}I_{B}$$

$$\frac{dR_{A}}{dt}= \gamma_{I}I_{A}$$

$$\frac{dR_{B}}{dt}= \gamma_{I}I_{B}$$

where *N_A_* and *N_B_* are the number of individuals in populations A and B respectively, *β_A_* and *β_B_* are the overall contact rates of populations A and B respectively times the probability of infection given contact, *γ_I_* is the rate of recovery. *ε* is the fraction of the total contacts made by individuals in a population that occurs with individuals in the other population and it describes the interaction strength between the two populations. $\varepsilon$ can take on values between 0 and 1, where *ε=*0 indicates that all interactions occur within each population and none between populations, and *ε=*1 indicates that all interactions occur between the populations and none within populations. The base parameters are set at *N_A_*=*N_B_*=5000, *γ_I_*=0.18 inverse days, $R_{0}^{A}=R_{0}^{B}=\frac{\beta_{A}}{\gamma_{I}}=\frac{\beta_{B}}{\gamma_{I}}=1.5$, which represent an outbreak similar to COVID-19, but the parameters are varied in sensitivity analyses. The initial condition was a single infected individual in population A (*I_A_*=1) and all other individuals were susceptible (*S_A_*=*N*-1, *S_B_*=*N*, *I_B_*=0, *R_A_*=0, *R_B_*=0). The differential equations are solved deterministically using the integrate.odeint function in the scipy package (version 1.6.2) in Python using a timestep of 0.1 days.

The concentration *C* of the pathogen in wastewater is given by

$$C=\frac{n_{A}+n_{B}}{v_{A}+v_{B}}$$

where *n_A_* and *n_B_* are the number of copies of pathogen genetic material (DNA or RNA) shed per day by population A and B, respectively, into the sampled wastewater and *v_A_* and *v_B_* are the volume of wastewater produced per day by population A and B, respectively, into the sampled wastewater.

The number of copies of pathogen genetic material, *n_A_* and *n_B_*, shed per day by population A and B, respectively, into the sampled wastewater is given by

$$n_{A}=nI_{A}f_{A}$$

$$n_{B}=nI_{B}f_{B}$$

Where *n* is the number of copies of pathogen genetic material shed per infected person per day (assumed to be the same for all infected individuals) and *f_A_* and *f_B_* are the fraction of total shed pathogen genetic material by population A and B respectively that is sampled by wastewater (determined by the sewer connectivity of each of the populations).

The volume of wastewater, *v_A_* and *v_B_*, produced per day by population A and B, respectively, into the sampled wastewater is given by

$$v_{A}=vN_{A}f_{A}$$

$$v_{B}=vN_{B}f_{B}$$

where *v* is the volume of wastewater produced per person per day (assumed to be the same for all individuals). The value used for *n* was $5\times{10}^{6.7}$ gene copies/person/day (estimate taken from Ref. [22] by multiplying ${10}^{4.7}$ gene copies/mL feces by 500 mL feces/day/person) and for *v* was 20 liters wastewater/person/day (per person daily wastewater usage approximated from per household daily wastewater usage from Ref. [23] assuming 4 people per household).

In addition to the assumptions mentioned above, the model makes the following additional assumptions: The probability of an infected individual shedding pathogen genetic material to wastewater is 1. Given that there is pathogen genetic material present in wastewater, the probability of detecting it is 1. Shedding of pathogen genetic material occurs only when an individual is infected. There are no stormwater contributions to wastewater to dilute the pathogen concentration.

To calculate the time of the maximum number of infections in a population and the time of the maximum concentration of pathogen gene content in wastewater data, we numerically calculated the time derivative at the midpoints of the time points as the difference between consecutive datapoints divided by the time interval and took the first time point at which the derivative crossed 0.

**References**

1. U.S. Census Bureau. AHS 2021 National Public Use File. Available: https://www2.census.gov/programs-surveys/ahs/2021/AHS%202021%20National%20PUF%20v1.0%20CSV.zip

2. U.S. Census Bureau. AHS 2019 National Public Use File. Available: https://www2.census.gov/programs-surveys/ahs/2019/AHS%202019%20National%20PUF%20v1.1%20CSV.zip

3. U.S. Census Bureau. AHS 2019 Metropolitan Public Use File. Available: https://www2.census.gov/programs-surveys/ahs/2019/AHS%202019%20Metropolitan%20PUF%20v1.0%20CSV.zip

4. U.S. Census Bureau, Department of Commerce Department of Housing and Urban Development. 2019 AHS Integrated National Sample: Sample Design, Weighting, and Error Estimation. U.S. Census Bureau, Department of Commerce Department of Housing and Urban Development; 2020 Sep. Available: https://www2.census.gov/programs-surveys/ahs/2019/2019%20AHS%20National%20Sample%20Design,%20Weighting,%20and%20Error%20Estimation.pdf

5. U.S. Census Bureau, Department of Commerce Department of Housing and Urban Development. 2019 AHS Metropolitan Sample: Sample Design, Weighting, and Error Estimation. U.S. Census Bureau, Department of Commerce Department of Housing and Urban Development; 2020 Sep. Available: https://www2.census.gov/programs-surveys/ahs/2019/2019%20AHS%20Metropolitan%20Sample%20Design,%20Weighting,%20and%20Error%20Estimation.pdf

6. U.S. Census Bureau, Department of Commerce Department of Housing and Urban Development. 2021 AHS Integrated National Sample: Sample Design, Weighting, and Error Estimation. U.S. Census Bureau, Department of Commerce Department of Housing and Urban Development; 2022 Aug. Available: https://www2.census.gov/programs-surveys/ahs/2021/2021%20AHS%20National%20Sample%20Design,%20Weighting,%20and%20Error%20Estimation.pdf

7. U.S. Census Bureau. AHS 2013 National Public Use File. Available: https://www2.census.gov/programs-surveys/ahs/2013/AHS%202013%20National%20PUF%20v2.0%20CSV.zip

8. U.S. Department of Housing and Urban Development and U.S. Census Bureau. Appendix B: Sample Design and Weighting: 2013. U.S. Department of Housing and Urban Development and U.S. Census Bureau; 2013. Available: https://www2.census.gov/programs-surveys/ahs/2013/2013%20AHS%20National%20Sample%20Design%20and%20Weighting.pdf

9. U.S. Department of Housing and Urban Development and U.S. Census Bureau. 2013 AHS Definitions. In: 2013 AHS Definitions [Internet]. [cited 9 Feb 2023]. Available: https://www2.census.gov/programs-surveys/ahs/2013/2013%20AHS%20Definitions.pdf

10. Florida Department of Health. Onsite Sewage Locations in Florida - 2012. Available: https://fgdl.org/zips/metadata/xml/septic_jun12.xml

11. Florida Department of Environmental Protection. Onsite Sewage FAQ - Permitting. [cited 10 Feb 2023]. Available: https://floridadep.gov/water/onsite-sewage/content/onsite-sewage-faq-permitting

12. Florida Department of Health. Septic System Owner’s Guide. In: floridahealth.gov [Internet]. [cited 10 Feb 2023]. Available: https://www.floridahealth.gov/environmental-health/onsite-sewage/_documents/septic-folder-8x11-link2.pdf

13. Utah Department of Environmental Quality Water Quality. Municipal Wastewater Planning Program (MWPP). In: Municipal Wastewater Planning Program (MWPP) [Internet]. 3 Feb 2023 [cited 10 Feb 2023]. Available: https://deq.utah.gov/water-quality/municipal-wastewater-planning-program-mwpp

14. Campbell H. 2021 MWPP Survey. In: 2021 MWPP Survey [Internet]. 27 Oct 2021 [cited 10 Feb 2023]. Available: https://documents.deq.utah.gov/water-quality/engineering/municipal-wastewater-planning-program/DWQ-2021-026722.pdf

15. Minnesota Pollution Control Agency. 2021 Wastewater Infrastructure Needs Instructions and Frequently Asked Questions (FAQ). Apr 2021 [cited 31 Mar 2023]. Available: https://www.pca.state.mn.us/sites/default/files/wq-wwtp3-06.pdf

16. Robinson C. 2017 SSTS Annual Report Subsurface Sewage Treatment Systems in Minnesota. Minnesota Pollution Control Agency; 2018 Apr. Available: https://www.pca.state.mn.us/sites/default/files/wq-wwists1-58.pdf

17. Environmental Protection Agency. Clean Watersheds Needs Survey 2012 Report to Congress. Environmental Protection Agency; 2016. Available: https://www.epa.gov/sites/default/files/2015-12/documents/cwns_2012_report_to_congress-508-opt.pdf

18. U.S. Environmental Protection Agency. 2012 Clean Watersheds Needs Survey. Available: https://ordspub.epa.gov/ords/cwns2012/f?p=cwns2012:25

19. Environmental Protection Agency. Clean Watersheds Needs Survey Detailed Scope and Methods. Environmental Protection Agency; 2012. Available: https://www.epa.gov/sites/default/files/2015-10/documents/cwns_2012_detailed_scope_and_methods-508.pdf

20. U.S. Census Bureau. 2020 Island Areas Censuses (IAC) Demographic Profile Summary File. U.S. Census Bureau; 2022 Oct. Available: https://www2.census.gov/programs-surveys/decennial/2020/technical-documentation/island-areas-tech-docs/demographic_profile/2020-iac-dpsf-technical-documentation.pdf

21. US Census Bureau. American Community Survey Information Guide. [cited 5 Jan 2023]. Available: https://www.census.gov/programs-surveys/acs/library/information-guide.html

22. Weidhaas J, Aanderud ZT, Roper DK, VanDerslice J, Gaddis EB, Ostermiller J, et al. Correlation of SARS-CoV-2 RNA in wastewater with COVID-19 disease burden in sewersheds. Sci Total Environ. 2021;775: 145790.

23. United States Environmental Protection Agency. WaterSense Statistics and Facts. 11 May 2022 [cited 3 Apr 2023]. Available: https://www.epa.gov/watersense/statistics-and-facts
