# Supplementary material for: Assessment of sewer connectivity in the United States and its implications for equity in wastewater-based epidemiology
Source: PLOS Glob Public Health. 2024 Apr 17;4(4):e0003039. doi: 10.1371/journal.pgph.0003039 (PMC11023481; doi:10.1371/journal.pgph.0003039)
Supplement: S2 Appendix — Compilation of data, assessment of data completeness, sewer connectivity in U.S. Island Areas, supplementary figures, and supplementary tables. (DOCX) [file pgph.0003039.s002.docx]

**S2 Appendix. Supplementary analyses.**

**Compilation of data and assessment of data completeness**

As the AHS does not provide finer scale geographic information than the census division for households outside of the 35 selected large metropolitan areas, we used the American Community Survey, which reports the percentage of occupied housing units lacking complete plumbing facilities by county and which allowed us to define the lower bound on the percentage of the population not connected to sewers. Additionally, we identified several state-level datasets, including locations of permitted septic tanks in Florida, sewer locations and populations served in Utah and Minnesota, and the 2012 EPA Clean Watersheds Needs Survey of sewer locations and populations served for 49 states (South Carolina did not participate). However, many of these state-level datasets are incomplete or potentially biased and thus only allow qualitative analyses. Finally, the 2020 US Census’ Island Areas Decennial survey provided information the percentage of households connected to sewer by Census Designated Place in the island areas of American Samoa, Guam, the Northern Mariana Islands, and the Virgin Islands (see **Table 1** and **S1 Table** for a summary of datasets).

We checked the consistency of the Florida Department of Health onsite sewage treatment and disposal systems inspections data reported in 2012 with other sources. We noted that an estimated 7% of households across the state were connected to septic tanks, but that this was substantially less than the Florida Department of Environmental Protection’s estimate that approximately one third of Florida’s population uses septic tanks [1] even after accounting for potential correlations of septic usage with household size (approximate increase in household size of 10% for a 50% increase in septic connectivity (**S7 Fig**) cannot explain the almost 5 time difference in septic connectivity across the two sources), suggesting that more households may be on septic tanks, and thus less on sewer systems. This discrepancy may exist because not every septic tank had a permit recorded in the state system, because there were changes since 2012, or because of uncertainty in the Florida Department of Environmental Protection’s estimate. Thus, we focused our analysis on relative comparisons across county subdivisions (**Fig 4**).

The 2012 EPA Clean Watersheds Needs Survey dataset includes voluntary submissions of the locations of publicly owned wastewater collection and treatment facilities and their estimated population served. The following states had among the highest reported needs and survey participation level (pg. 6 and Table A-1 of Ref. [2]) and were the focus of our analyses: New York, California, Florida, New Jersey, Maryland, Iowa, Minnesota, and Michigan. Comparison of Florida in the EPA and Florida Department of Public Health datasets showed consistent spatial trends (less connection to sewers and more connection to septic systems in the panhandle and inland) (**Figs 4** and **S4**). Additionally, in both datasets, counties with more connection to sewers are more Asian (EPA: Pearson r = 0.53 (p = 4.2$\times$10^-4^), Florida Department of Public Health: Pearson r = 0.31 (p = 3.2$\times$10^-7^)). Comparison of Minnesota in the EPA dataset, Minnesota Wastewater Infrastructure Needs Survey, and Minnesota SSTS datasets also showed consistent spatial trends (more connection to sewers and less connection to subsurface sewage treatment systems around Minneapolis) (**S14 Fig**). We found a significant correlation between the AHS and EPA datasets for the largest 35 metropolitan areas in the states with good data collection (**S15 Fig**), despite deviations from a one-to-one correspondence suggesting overall systematic biases in the data.

The 2021 Utah Municipal Wastewater Planning Survey estimated the populations receiving collection by municipal utilities that owned or operated a sanitary sewerage system. The purpose of the survey is to aid in overseeing and communicating with the wastewater industry in Utah. We noticed that these data may have not be quantitatively accurate but are likely qualitatively informative. First, large swaths of the state appeared not to receive wastewater collection (**S16 Fig**), but this may partly reflect that only 71% of contacts responding to the survey [3]. Second, in some county subdivisions, >100% of the population appeared connected to sewers, likely due to errors in estimation, differences in how combined treatment and collection facilities reported population estimates, or collection populations resided in neighboring county subdivisions. While the 5 county subdivisions with the highest percentages of American Indian and Alaska Natives (~20-100%) were reported to not be connected to sewers at all in this dataset, we believe this is because Indian reservations are not included in the dataset (as they are under federal, not state jurisdiction). In fact, all 5 of these counties (Uintah and Ouray, Casa del Eco Mesa-White Mesa, West Juab, Oljato, Blanding) include Indian reservations. Additionally, the county subdivision with the highest percentage of Black or African American; Native Hawaiian and Other Pacific Islander; or Hispanic are in county subdivisions has nobody receiving collection (**S17 Fig**); however, we cannot rule out that this is due to underreporting from these county subdivisions.

The 2021 Minnesota Wastewater Infrastructure Needs Survey reported whether communities have a collection system. The data from this survey was geographically sparse, as communities (the level of aggregation of the dataset) often directly mapped to a census designated place (CDP), and CDPs do not cover the entire state (**S14**a **Fig**). Consequently, the sewer connectivity of the rural areas between census designated places could not be determined from this dataset. Additionally, not all communities responded to the survey. Of the 523 communities that participated in the survey, all but 11 had a collection system. The geographic distribution of sewer connectivity qualitatively matched that of the 2017 Subsurface Sewage Treatment Systems (SSTS) Annual Report of the number of reported subsurface sewage treatment systems (septic tanks) by county in Minnesota (**S14**b **Fig**); however, 7 out of the 218 contacted local government units did not respond, 14 reported having zero SSTS within their jurisdiction despite permitting SSTS and not all SSTS programs were able to be identified to be contacted.

Given the data incompleteness and biases for the Utah and Minnesota datasets, we excluded them from the analyses in the main text.

**Sewer connectivity in the U.S. Island Areas**

From the 2020 US Census Island Areas Decennial Survey of household characteristics, the overall levels of household sewer connection in the island areas were lower than in the states (**S18 Fig**). In Guam, the Northern Mariana Islands, the Virgin Islands, and American Samoa, the overall connection to sewer systems across each island was 65%, 51%, 66%, and 52% of households, respectively (compared to an overall 83% connection across mainland US). To ask whether there was strong spatial variability across each island, we looked at the median household connection by Census Designated Place (CDP). A median of 64%, 32%, 70%, and 13% households by CDP in Guam, the Northern Mariana Islands, the Virgin Islands, and American Samoa, respectively, were connected to sewers. This suggests that sewer connectivity is particularly spatially concentrated in the Northern Mariana Islands and American Samoa (difference in median household connection by CDP and overall island connection), although all island areas showed some spatial variability in connectivity when visually assessing maps (**S19 Fig**). The only significant correlation of sewer connectivity with demographic or economic characteristics was in American Samoa, where CDPs with a higher percentage of Black or African Americans were more connected to sewers (Pearson r = 0.38, q-value = 0.01).

**References**

1. General Facts and Statistics about Wastewater in Florida. [cited 10 Feb 2023]. Available: https://floridadep.gov/water/domestic-wastewater/content/general-facts-and-statistics-about-wastewater-florida

2. Environmental Protection Agency. Clean Watersheds Needs Survey 2012 Report to Congress. Environmental Protection Agency; 2016. Available: https://www.epa.gov/sites/default/files/2015-12/documents/cwns_2012_report_to_congress-508-opt.pdf

3. Campbell H. 2021 MWPP Survey. In: 2021 MWPP Survey [Internet]. 27 Oct 2021 [cited 10 Feb 2023]. Available: https://documents.deq.utah.gov/water-quality/engineering/municipal-wastewater-planning-program/DWQ-2021-026722.pdf
