# Supplementary figures and images for: Assessment of sewer connectivity in the United States and its implications for equity in wastewater-based epidemiology
Source: PLOS Glob Public Health. 2024 Apr 17;4(4):e0003039. doi: 10.1371/journal.pgph.0003039 (PMC11023481; doi:10.1371/journal.pgph.0003039)

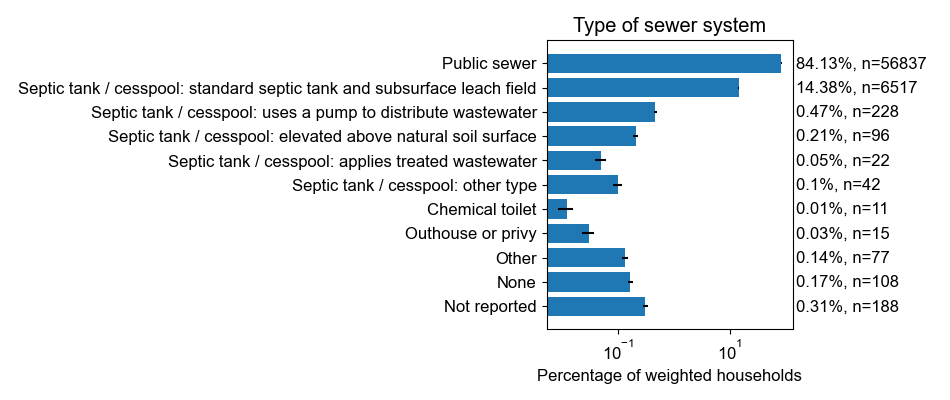

Supplement: S1 Fig — The number of households sampled (n) for each category are also shown. Data are from 2021 American Housing Survey. (PNG) [file pgph.0003039.s003.png]

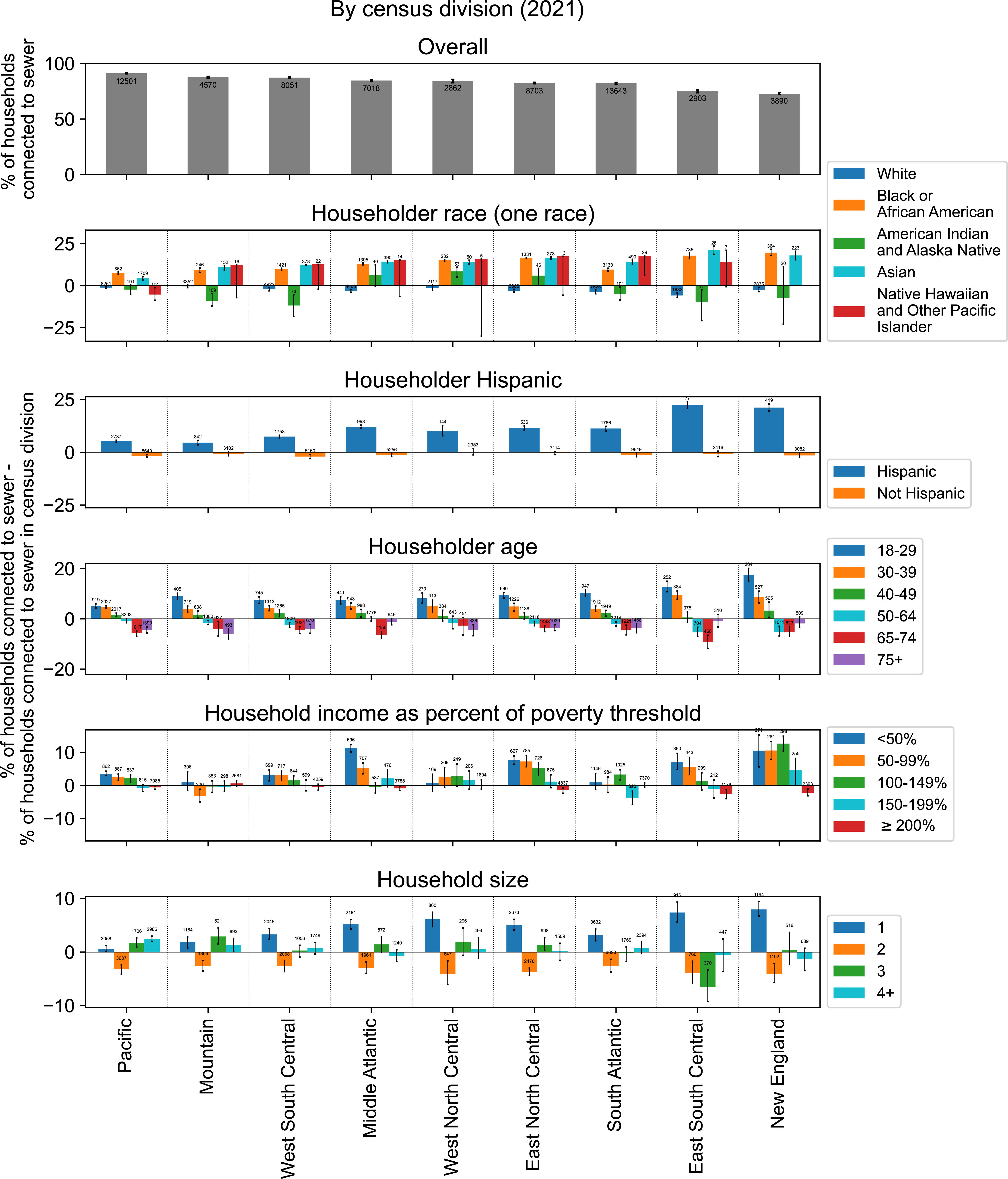

Supplement: S2 Fig — (PNG) [file pgph.0003039.s004.png]

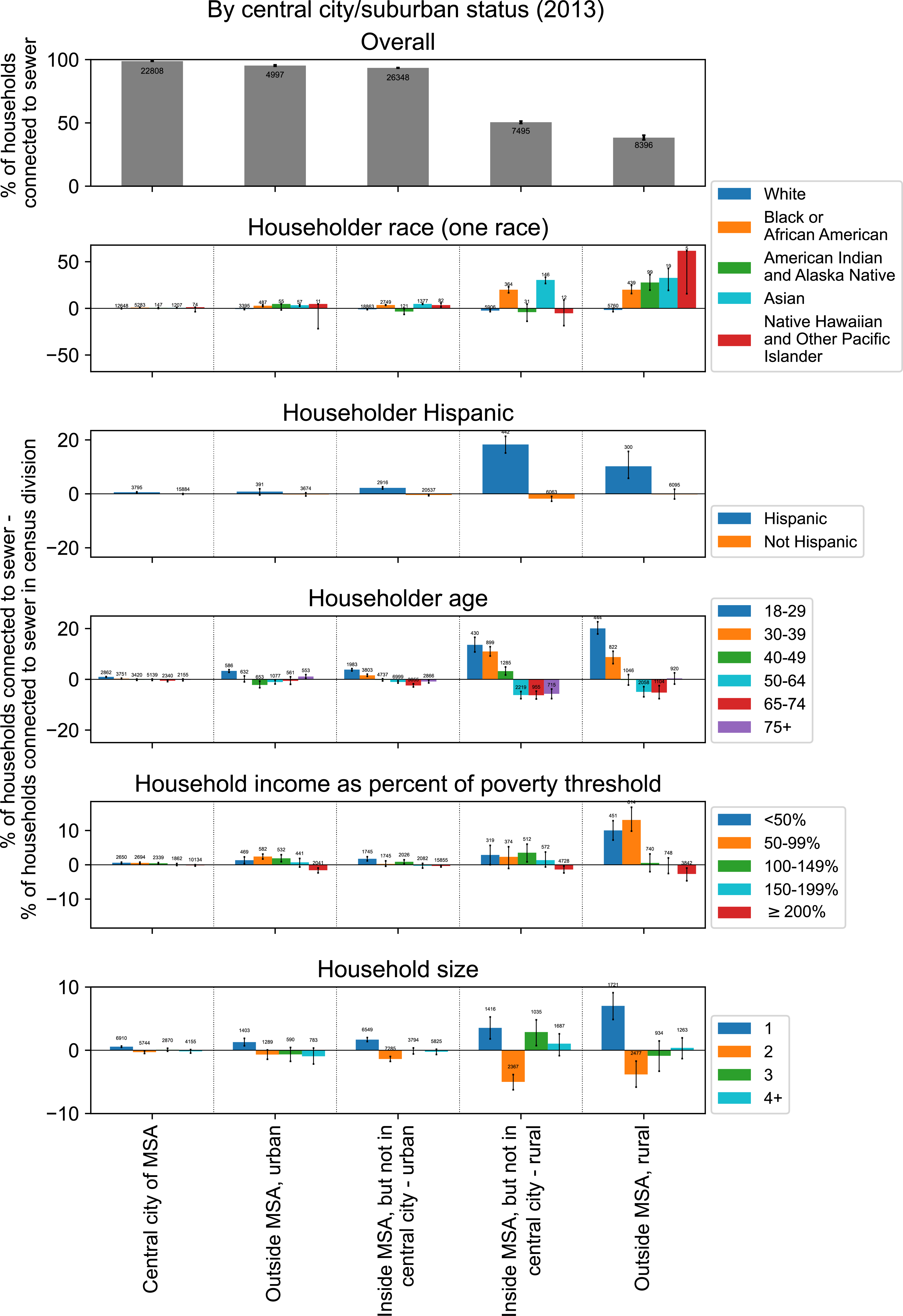

Supplement: S3 Fig — (PNG) [file pgph.0003039.s005.png]

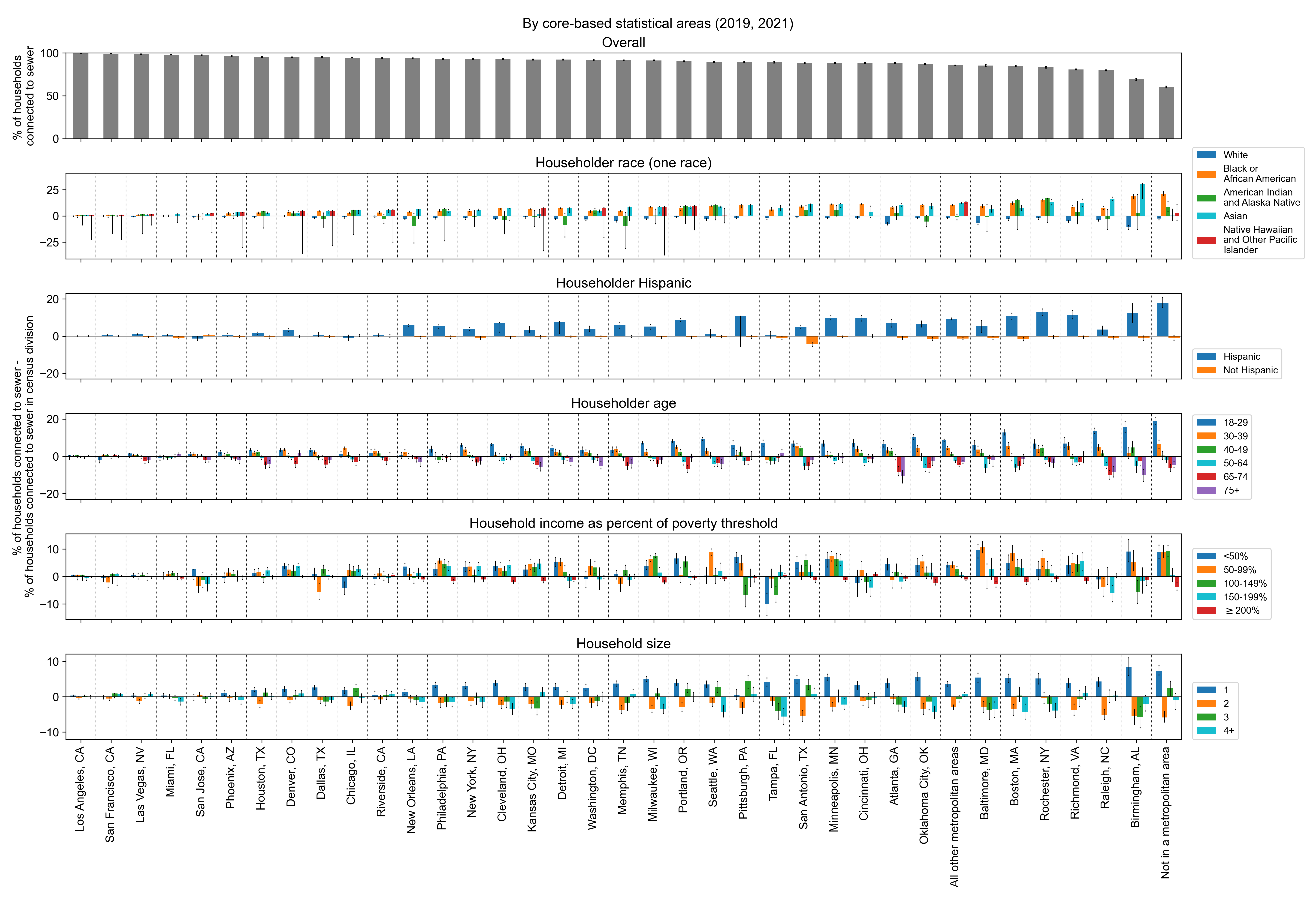

Supplement: S4 Fig — Data are from the 2019 and 2021 U.S. Census American Housing Survey [12, 23]. The number of sampled households for each category can be found in the Github repository in outputs/ahs_sewer_connectivity_by_cbsa.csv in the “num_observations” column. (PNG) [file pgph.0003039.s006.png]

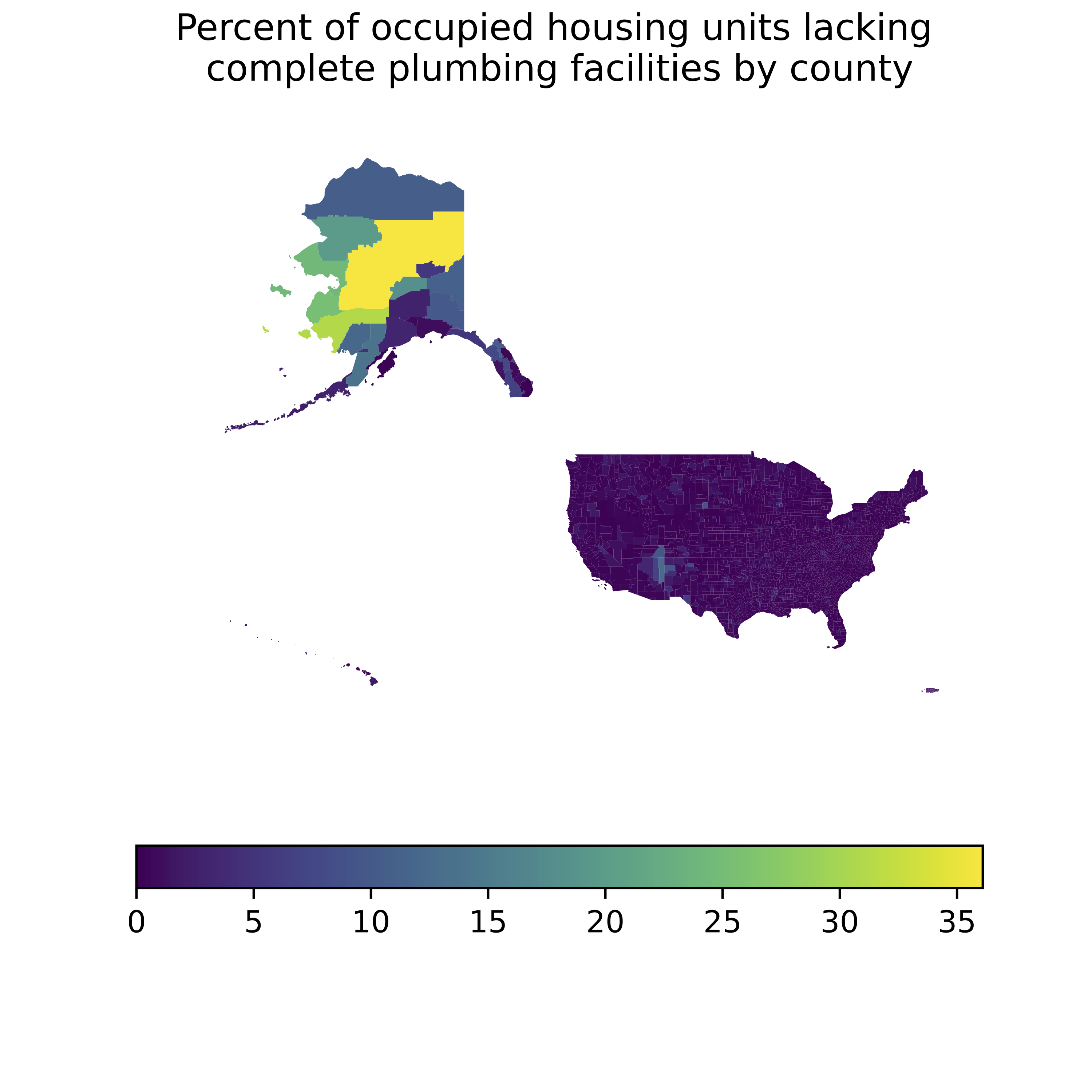

Supplement: S5 Fig — Data are from the 2021 U.S. Census American Community Survey. The map base layer is taken from the U.S. Census 2021 TIGER/Line Shapefile by U.S. County (https://www2.census.gov/geo/tiger/TIGER2021/COUNTY/tl_2021_us_county.zip; terms of use: https://www2.census.gov/geo/pdfs/maps-data/data/tiger/tgrshp2021/TGRSHP2021_TechDoc_Ch1.pdf). (PNG) [file pgph.0003039.s007.png]

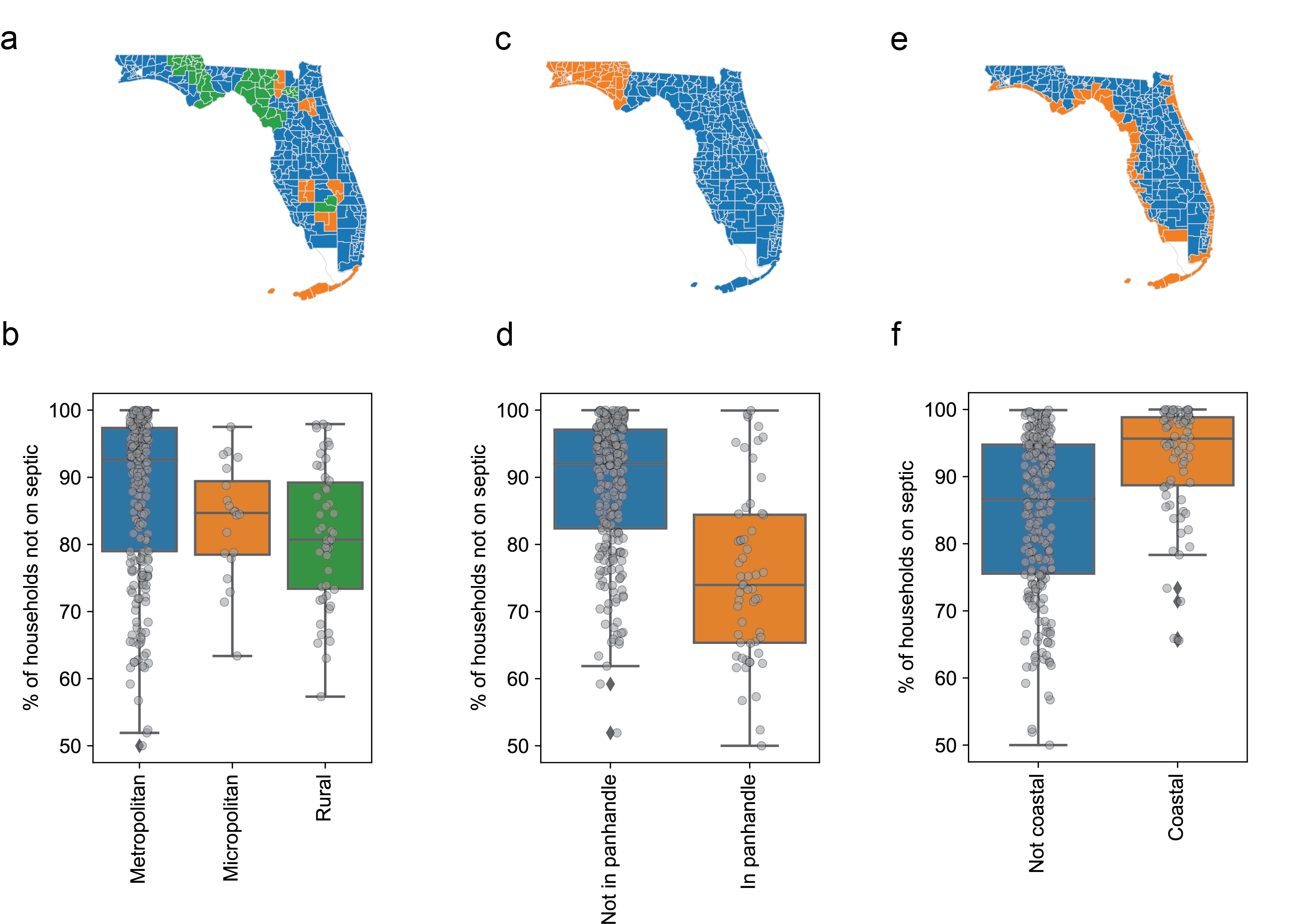

Supplement: S6 Fig — Data are from the Florida Department of Health septic tank inspection permits reported in 2012. (a) Map of county subdivision colored by whether it is a Metropolitan Statistical Area (blue), Micropolitan Statistical Area (orange), or Rural Area (neither metropolitan not micropolitan, green). (b) Percentage of households not on septic in county subdivisions stratified by whether the county subdivision is a Metropolitan Statistical Area, Micropolitan Statistical Area, or Rural Area. County subdivisions that are Metropolitan Statistical Areas have a higher percentage of households not on sewer than those that are Micropolitan Statistical Areas, but the effect is not significant (p = 0.052). County subdivisions that are Micropolitan Statistical Areas have a higher percentage of households not on sewer than those that are Rural Areas, but the effect is not significant (p = 0.37). (c) Map of county subdivisions colored by whether they are always included in references to the Florida panhandle. Orange: county subdivisions in the panhandle. Blue: county subdivisions not in the panhandle. (d) Percentage of households not on septic in county subdivisions stratified by whether the county subdivision is in the panhandle. Each point represents a county subdivision. County subdivisions in the panhandle have a significantly lower percentage of households not on septic (p < 10−9). (e) Map of county subdivisions colored by whether they have a coastline. Orange: county subdivision with coastline. Blue: county subdivisions without coastline. (f) Percentage of households not on septic in county subdivisions stratified by coastal and not coastal county subdivisions. Each point represents a county subdivision. County subdivisions not on the coast have a significantly higher fraction of households on septic (p<10−9). The map base layers in (a), (c), and (e) are taken from the U.S. Census 2012 TIGER/Line Shapefile by Florida County Subdivision (https://www2.census.gov/geo/tiger/TIGER2012/C [file pgph.0003039.s008.png]

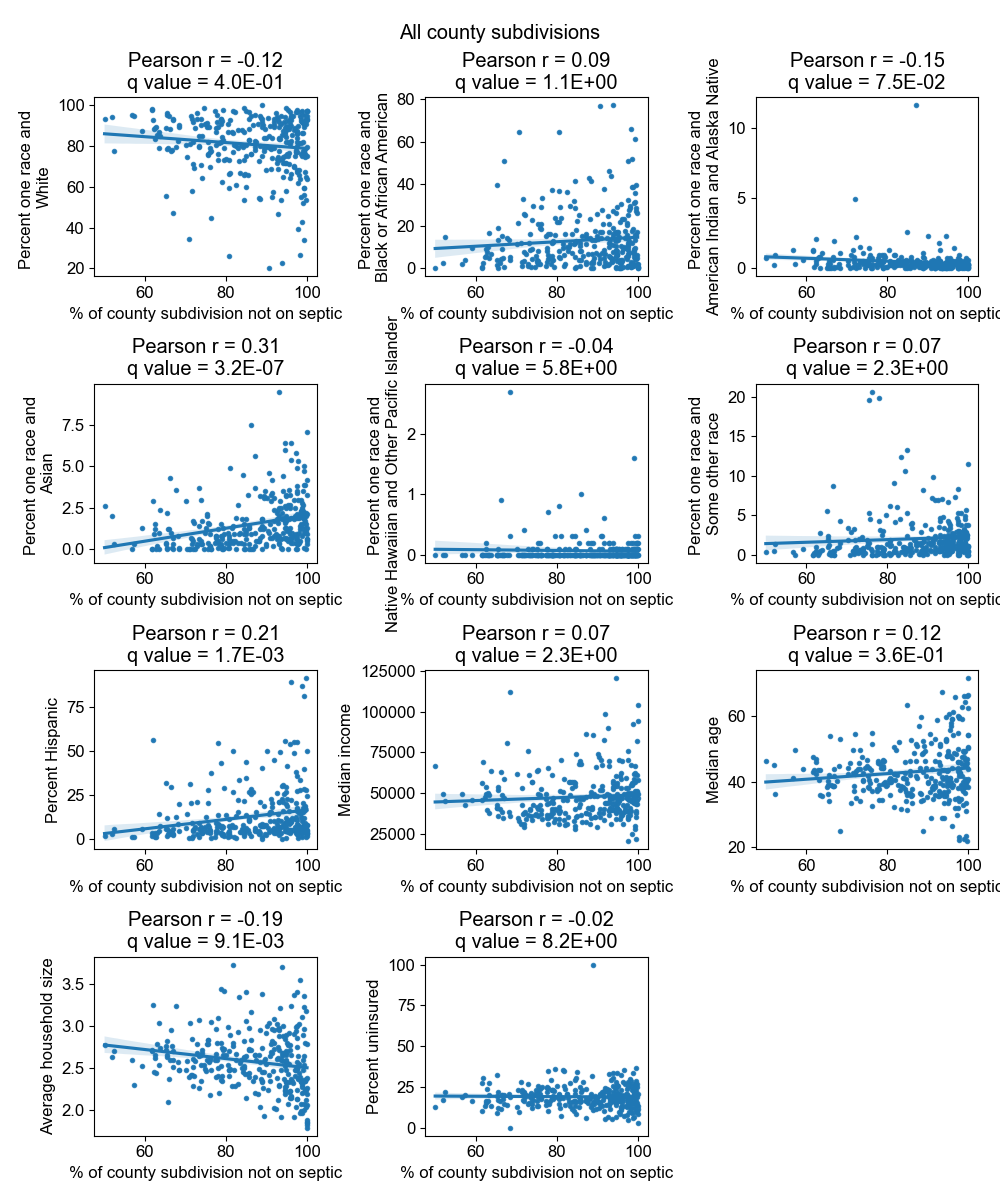

Supplement: S7 Fig — Data are from the Florida Department of Health septic tank inspection permits reported in 2012. Each point represents a county subdivision. Only county subdivisions with at least 5 households and 20 population size were included. (PNG) [file pgph.0003039.s009.png]

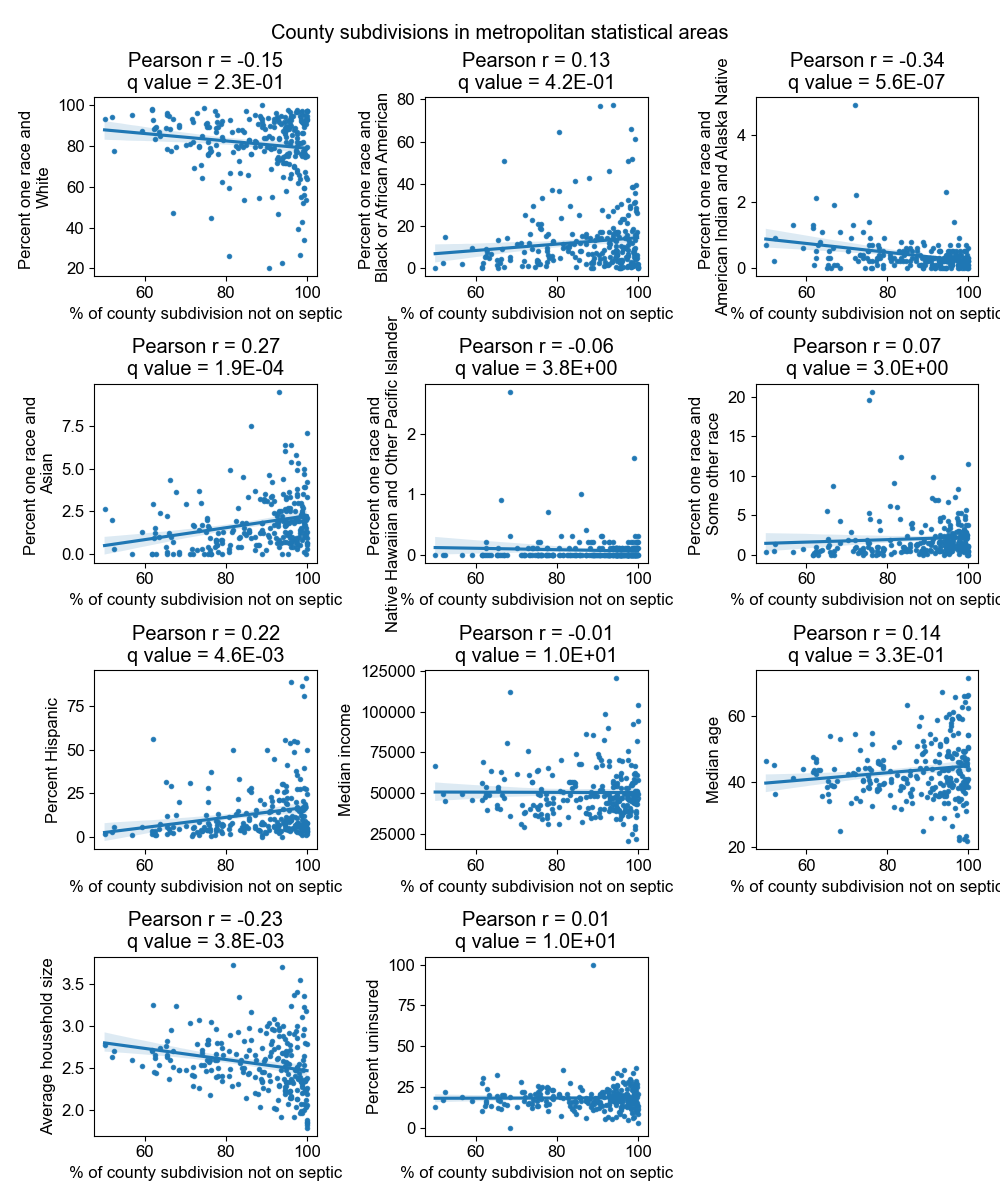

Supplement: S8 Fig — Data are from the Florida Department of Health septic tank inspection permits reported in 2012. Each point represents a county subdivision. Only metropolitan county subdivisions with at least 5 households and 20 population size were included. (PNG) [file pgph.0003039.s010.png]

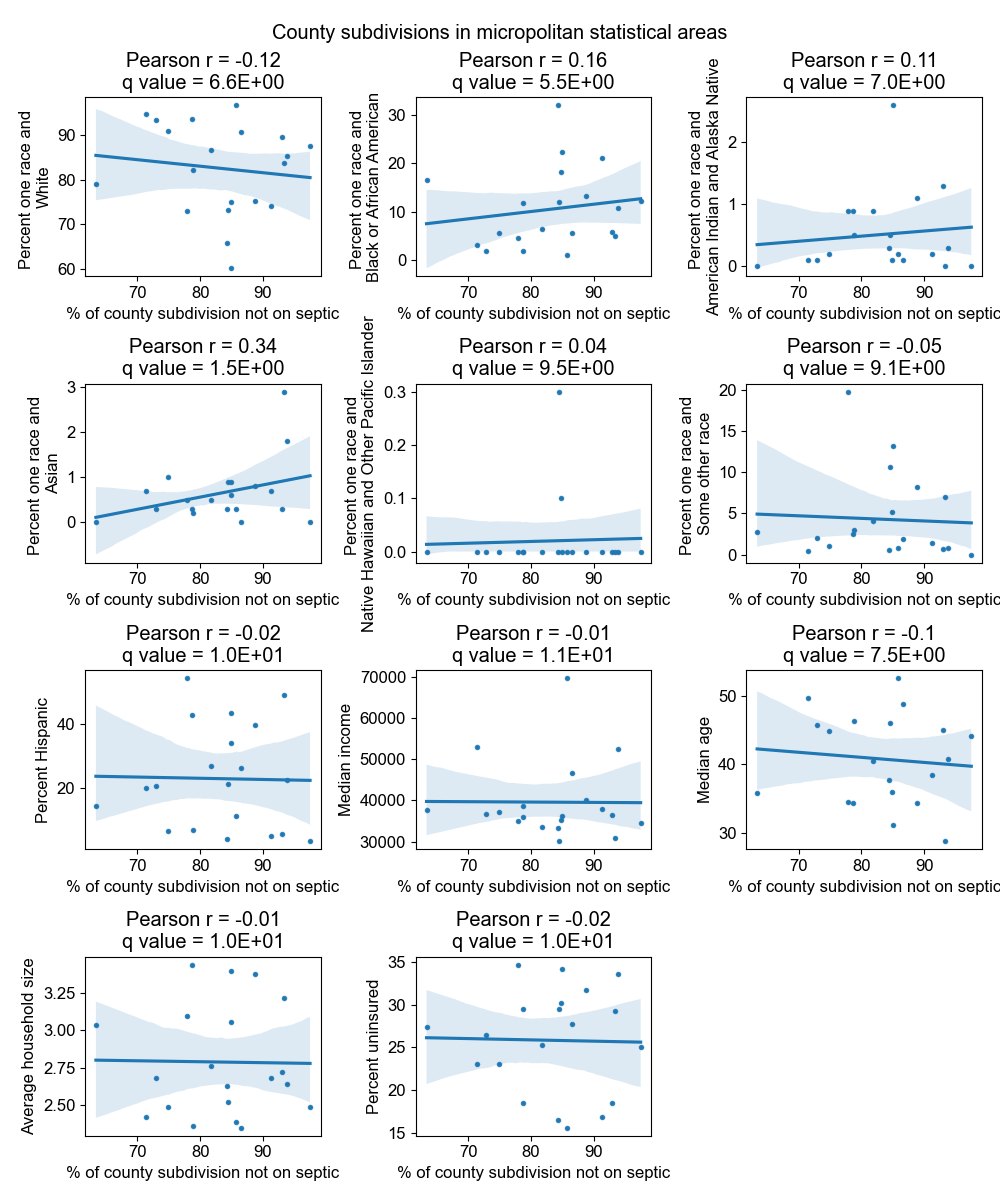

Supplement: S9 Fig — Each point represents a county subdivision. Data are from the Florida Department of Health septic tank inspection permits reported in 2012. Only micropolitan county subdivisions with at least 5 households and 20 population size were included. (PNG) [file pgph.0003039.s011.png]

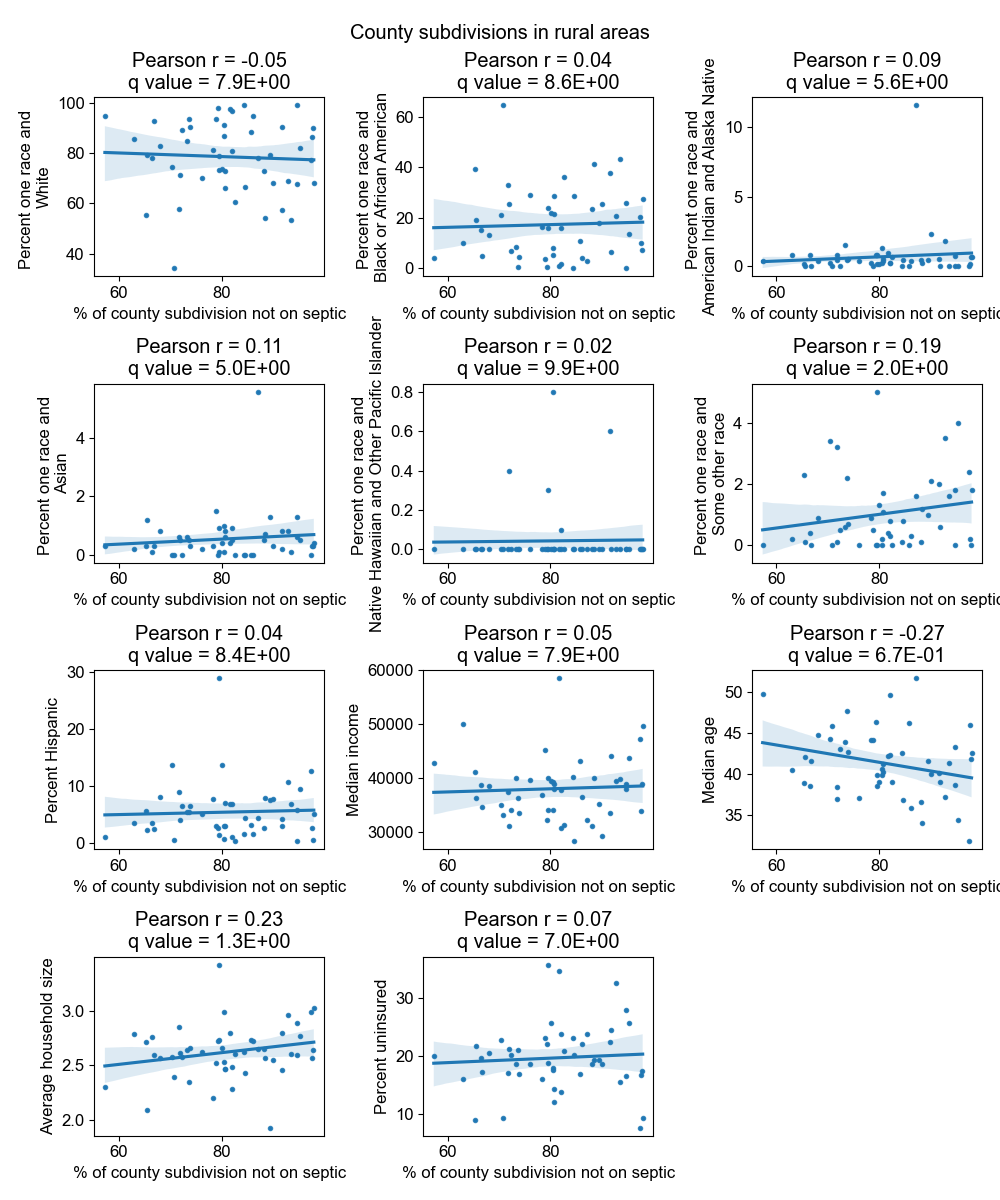

Supplement: S10 Fig — Data are from the Florida Department of Health septic tank inspection permits reported in 2012. Each point represents a county subdivision. Only rural county subdivisions with at least 5 households and 20 population size were included. (PNG) [file pgph.0003039.s012.png]

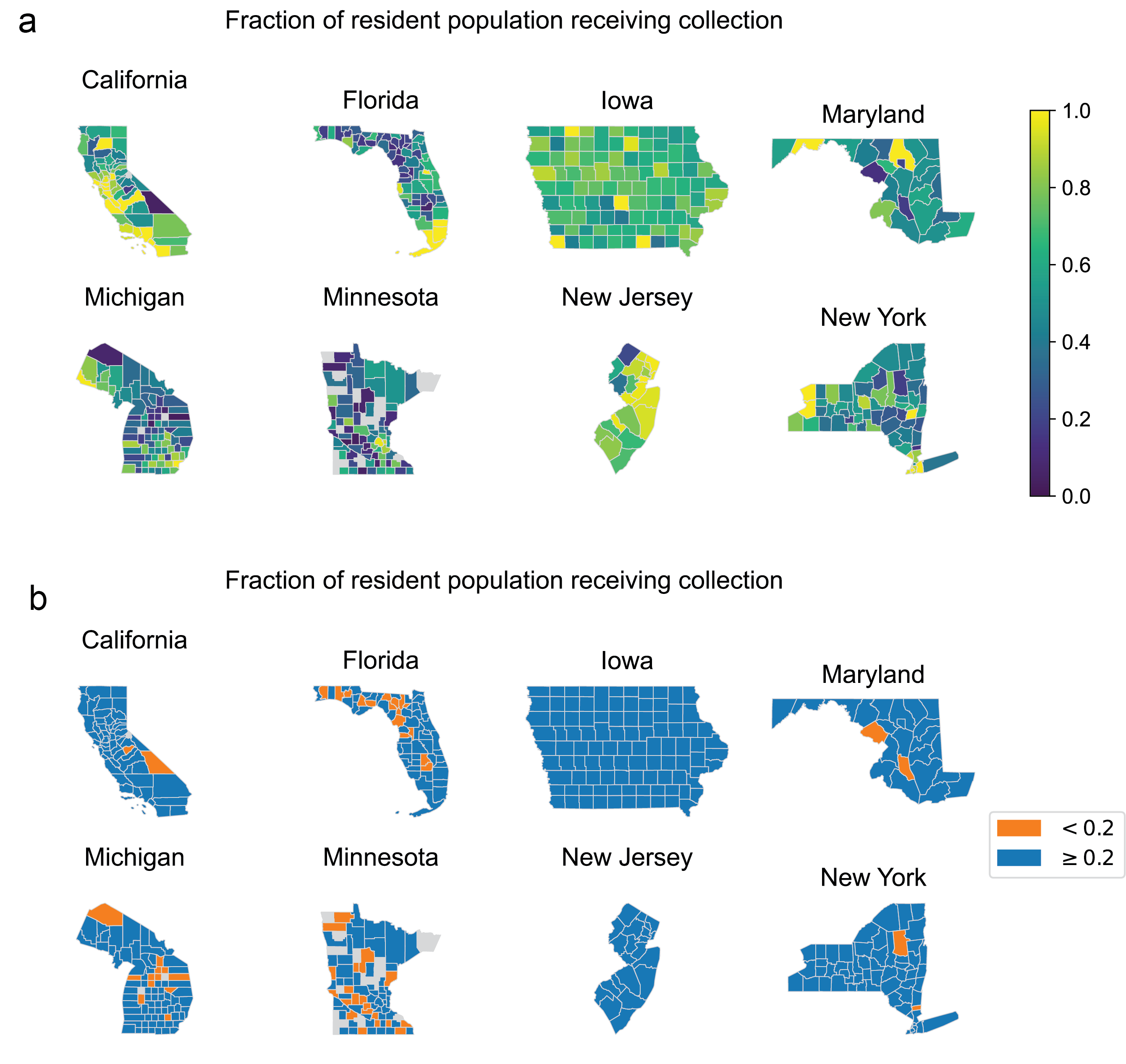

Supplement: S11 Fig — Data are from the 2012 EPA Clean Watersheds Needs Survey. (a) Fraction shown on a continuous scale. (b) Fraction above and below 0.2. Only states that had more comprehensive responses in the survey are shown. Counties that did not report any data in the survey are shown in gray. Counties than report more than 100% of the present resident population receiving collection are shown in (a) as 1 and in (b) as ≥0.2. The map base layer is taken from the U.S. Census 2012 TIGER/Line Shapefile by U.S. County (https://www2.census.gov/geo/tiger/TIGER2012/COUNTY/tl_2012_us_county.zip; terms of use: https://www2.census.gov/geo/pdfs/maps-data/data/tiger/tgrshp2012/TGRSHP2012_TechDoc_Ch1.pdf). (PNG) [file pgph.0003039.s013.png]

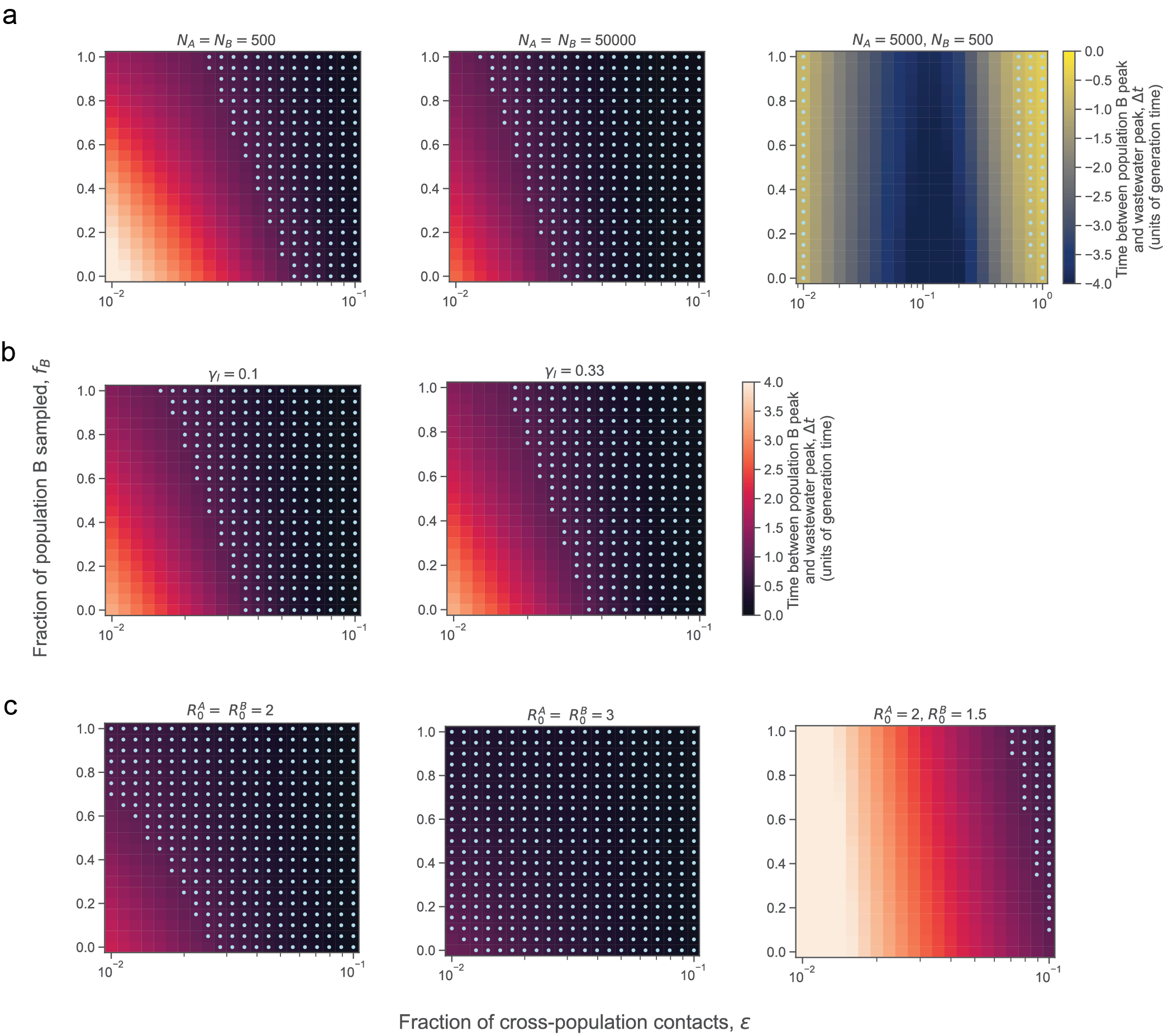

Supplement: S12 Fig — The effect on the time between population B peak and wastewater peak of varying the (a) population size of the two populations (NA, NB), (b) recovery rate (ƔI in units of inverse days), and (c) basic reproduction number of the two populations (R0A, R0B) across a range of values. Unless where indicated, the parameters were NA = NB = 5000, ƔI = 0.18 days-1, and R0A, R0B = 1.5. (PNG) [file pgph.0003039.s014.png]

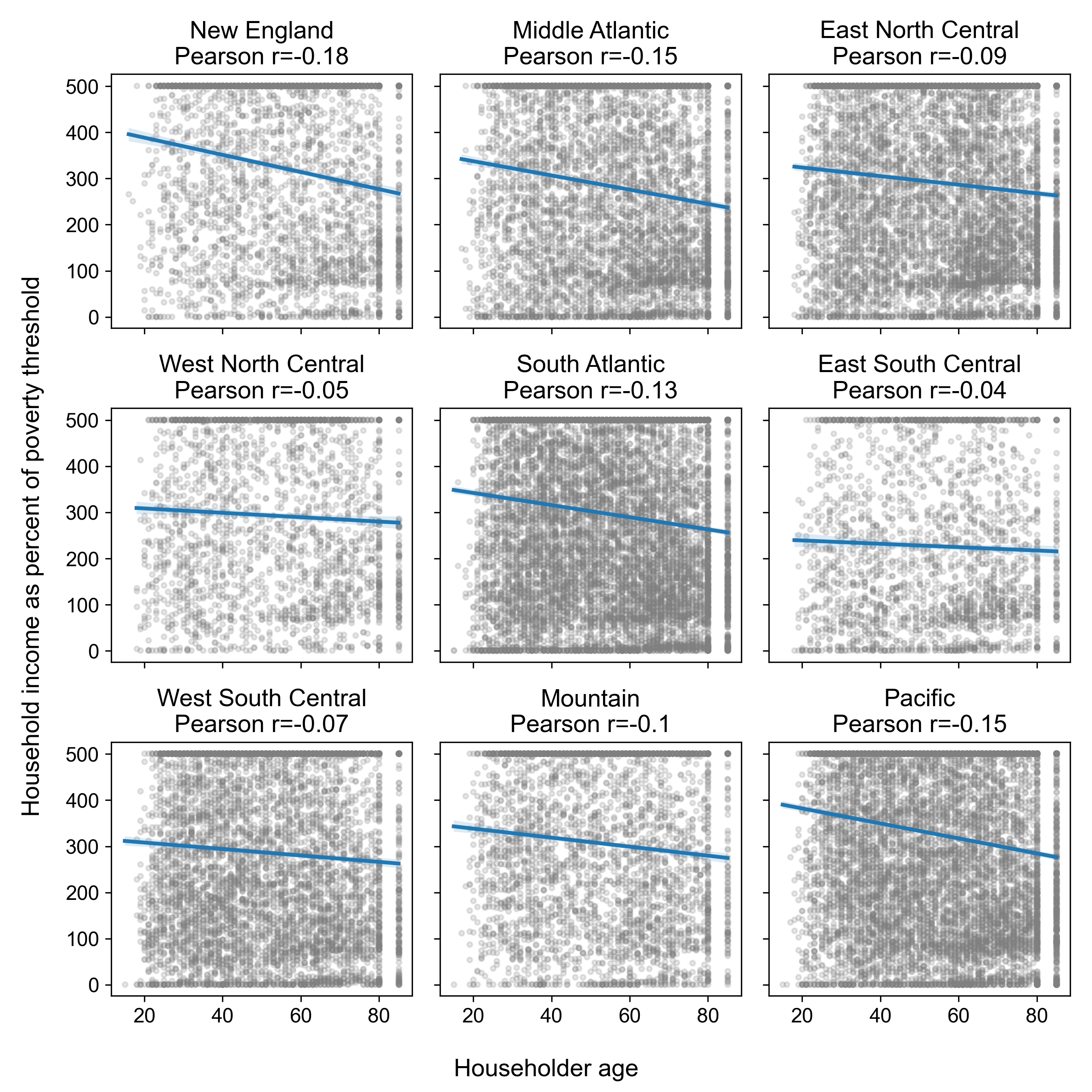

Supplement: S13 Fig — (PNG) [file pgph.0003039.s015.png]

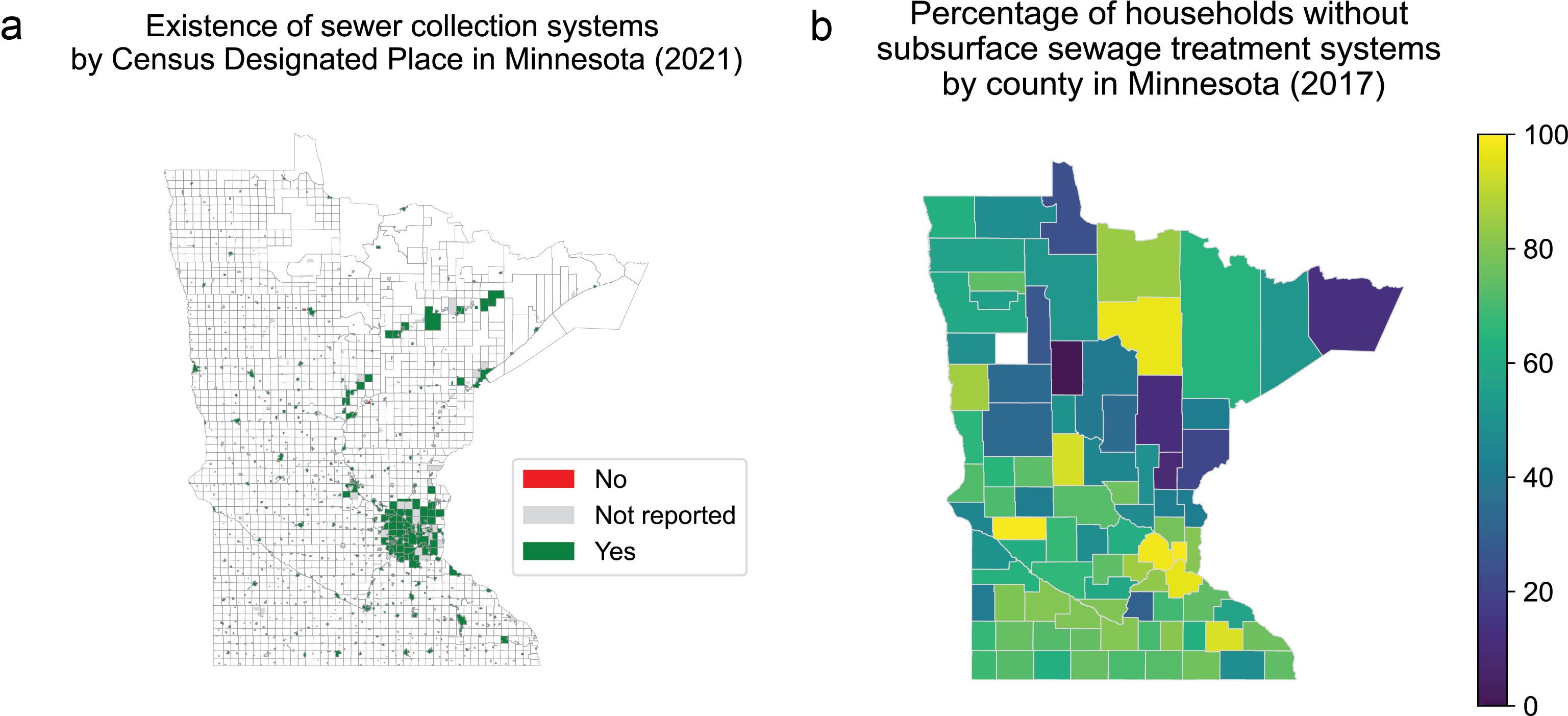

Supplement: S14 Fig — (a) Map showing whether a community in Minnesota (mapped to Census Designated Place) reported having a collection system in the 2021 Minnesota Wastewater Infrastructure Needs Survey. Almost all communities mapped to Census Designated Places. The map base layer is taken from the U.S. Census 2021 TIGER/Line Shapefile by Minnesota Census Designated Place (https://www2.census.gov/geo/tiger/TIGER2021/PLACE/tl_2021_27_place.zip; terms of use: https://www2.census.gov/geo/pdfs/maps-data/data/tiger/tgrshp2021/TGRSHP2021_TechDoc_Ch1.pdf). (b) Percentage of households without subsurface sewage treatment systems (i.e. without septic tanks, suggesting sewered) by county reported in the 2017 Subsurface Sewage Treatment Systems in Minnesota Annual Report [56]. Counties reporting more than 100% of households having subsurface sewage treatment systems are set at 100% (shown as 0% without subsurface sewage treatment systems in the map). The map base layer is taken from the U.S. Census 2017 TIGER/Line Shapefile by U.S. County (https://www2.census.gov/geo/tiger/TIGER2017/COUNTY/tl_2017_us_county.zip; terms of use: https://www2.census.gov/geo/pdfs/maps-data/data/tiger/tgrshp2017/TGRSHP2017_TechDoc_Ch1.pdf). (PNG) [file pgph.0003039.s016.png]

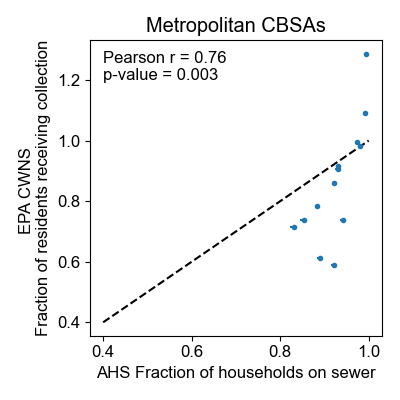

Supplement: S15 Fig — Census American Housing Survey (AHS) datasets in core based statistical areas (CBSA). Data from the EPA CWNS are of the fraction of residents in the CBSA receiving sewage collection. Data from the AHS are of the fraction of households in the CBSA on sewer. Only CBSAs that were oversampled in the AHS data and were in states that had more comprehensive responses in the EPA CWNS were included (see Methods). The dashed black line shows y = x as a reference. (PNG) [file pgph.0003039.s017.png]

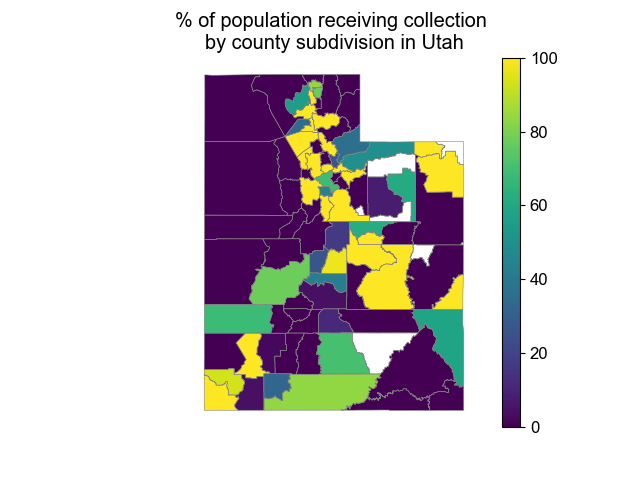

Supplement: S16 Fig — Data are from the 2021 Utah Municipal Wastewater Planning Survey. All values less than 0 are displayed as 0. County subdivisions with fewer than 5 households or 20 population size are displayed in white. Note that Indian reservations were not surveyed in this dataset. The map base layer is taken from the U.S. Census 2021 TIGER/Line Shapefile by Utah County Subdivision (https://www2.census.gov/geo/tiger/TIGER2021/COUSUB/tl_2021_49_cousub.zip; terms of use: https://www2.census.gov/geo/pdfs/maps-data/data/tiger/tgrshp2021/TGRSHP2021_TechDoc_Ch1.pdf). (PNG) [file pgph.0003039.s018.png]

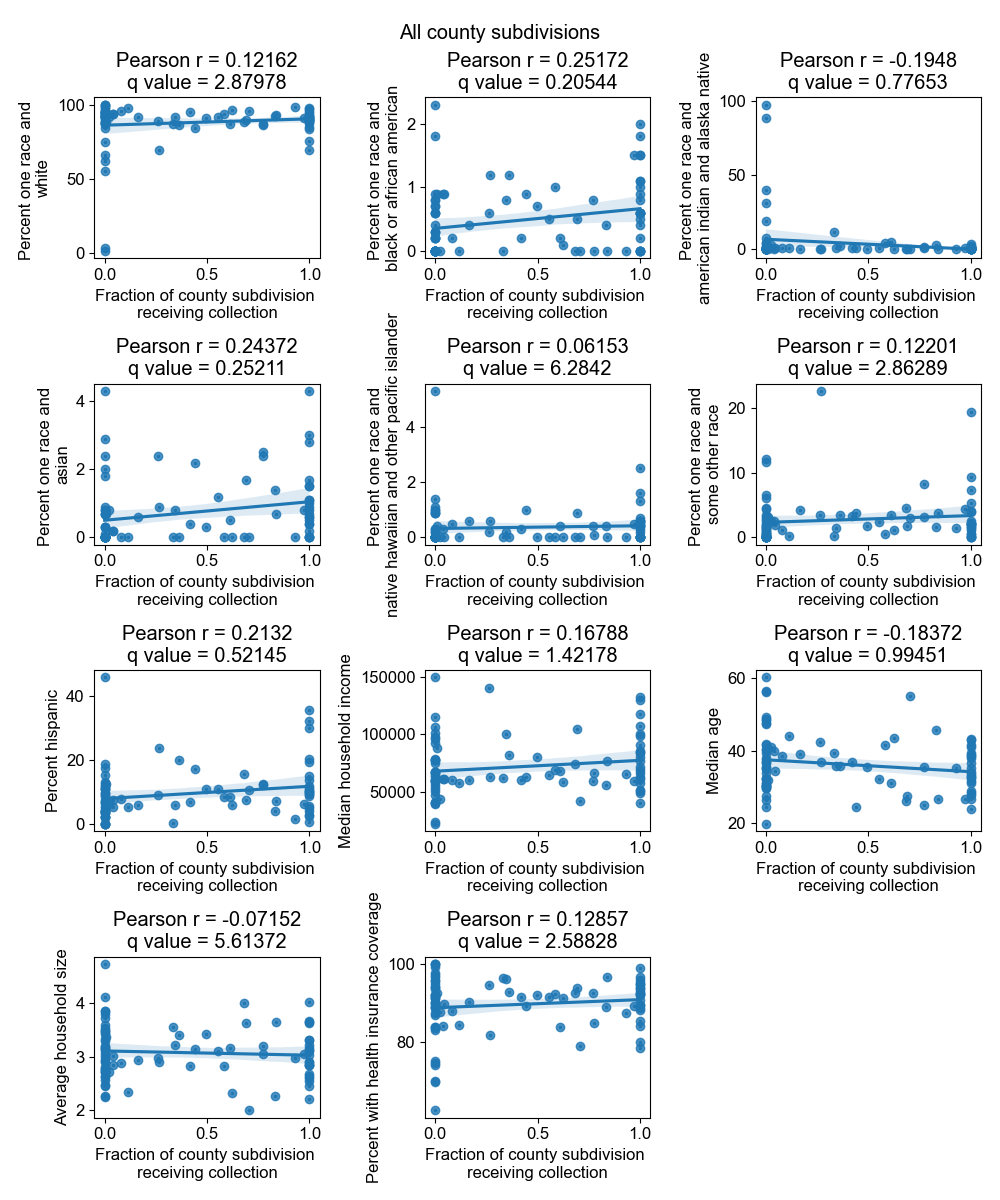

Supplement: S17 Fig — Data are from the 2021 Utah Municipal Wastewater Planning Survey. Note that Indian reservations were not surveyed in this dataset. Values of the fraction of the county subdivision receiving collection greater than 1 were set to 1. (PNG) [file pgph.0003039.s019.png]

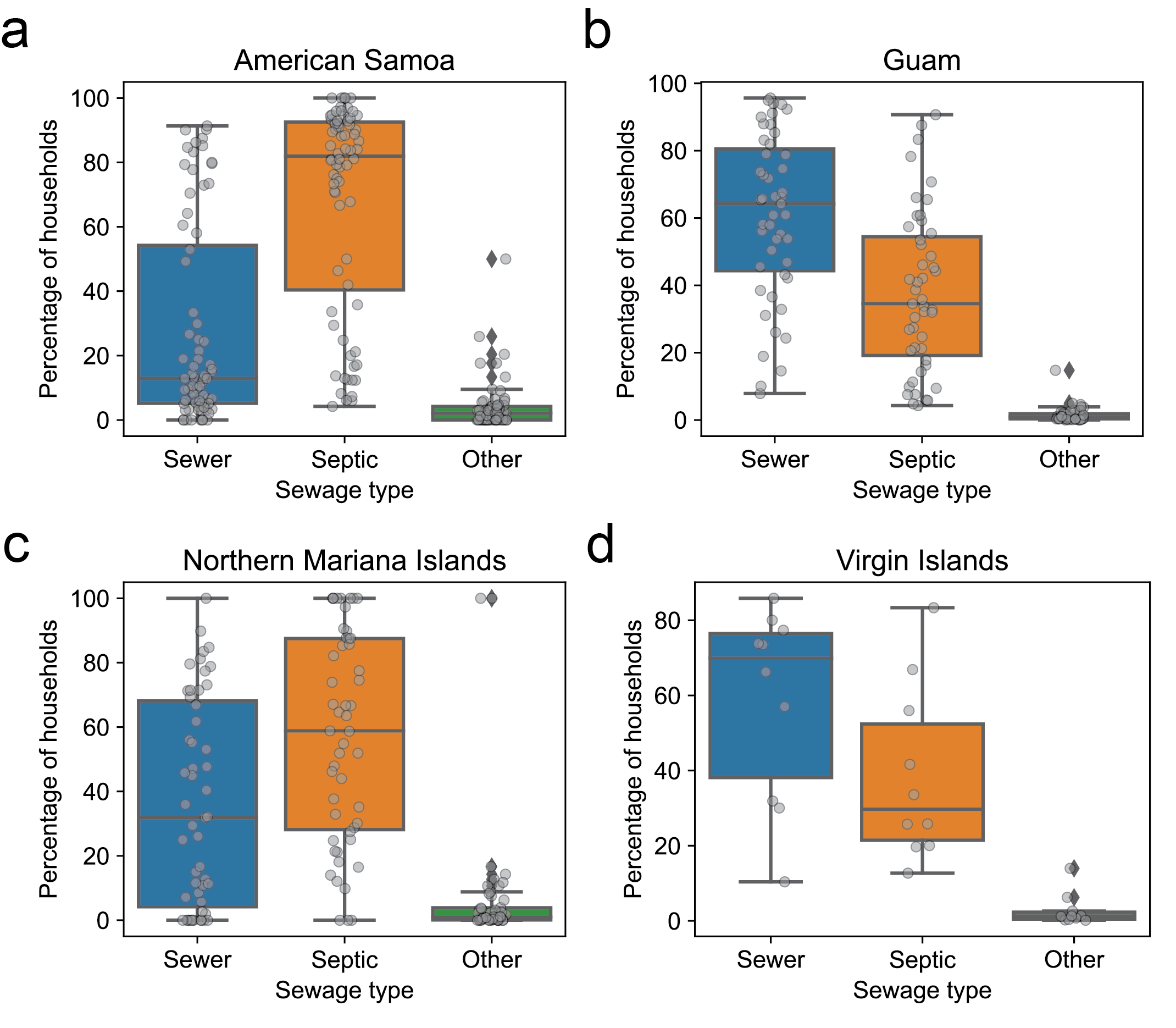

Supplement: S18 Fig — (a) American Samoa, (b) Guam, (c) the Northern Mariana Islands, and (d) the Virgin Islands. Data are from the 2020 U.S. Census Island Areas Decennial Survey. (PNG) [file pgph.0003039.s020.png]

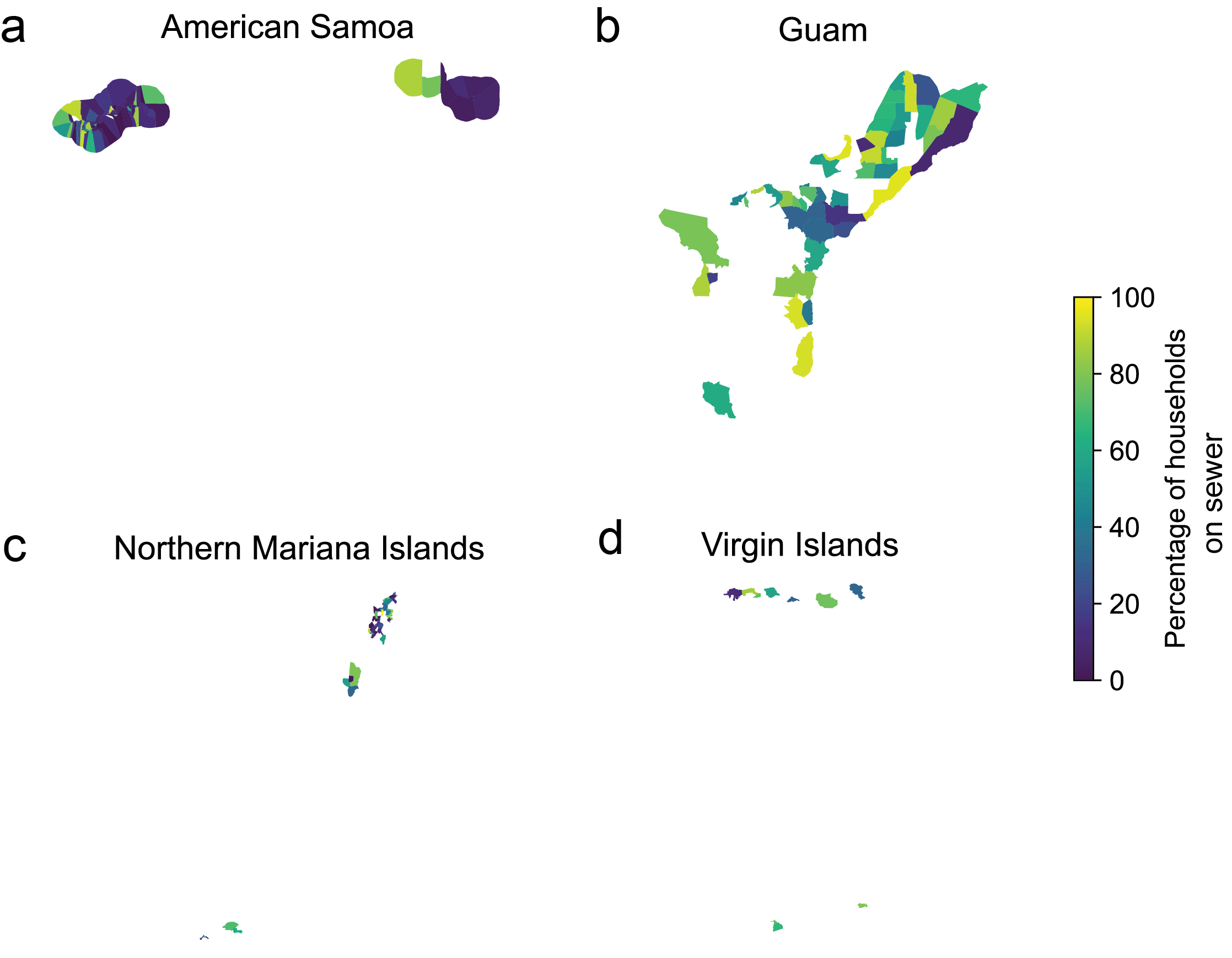

Supplement: S19 Fig — (a) American Samoa, (b) Guam, (c) the Northern Mariana Islands, and (d) the Virgin Islands. Data are from the 2020 U.S. Census Island Areas Decennial Survey. The map base layers are taken from the U.S. Census 2020 TIGER/Line Shapefile by American Samoa Census Designated Place (https://www2.census.gov/geo/tiger/TIGER2020/PLACE/tl_2020_60_place.zip), Guam Census Designated Place (https://www2.census.gov/geo/tiger/TIGER2020/PLACE/tl_2020_66_place.zip), Northern Mariana Islands Census Designated Place (https://www2.census.gov/geo/tiger/TIGER2020/PLACE/tl_2020_69_place.zip), and Virgin Islands Census Designated Place (https://www2.census.gov/geo/tiger/TIGER2020/PLACE/tl_2020_78_place.zip). The terms of use for all maps can be found at: https://www2.census.gov/geo/pdfs/maps-data/data/tiger/tgrshp2020/TGRSHP2020_TechDoc_Ch1.pdf. (PNG) [file pgph.0003039.s021.png]
