# Supplementary material for: Assessment of sewer connectivity in the United States and its implications for equity in wastewater-based epidemiology
Source: PLOS Glob Public Health. 2024 Apr 17;4(4):e0003039. doi: 10.1371/journal.pgph.0003039 (PMC11023481; doi:10.1371/journal.pgph.0003039)
Supplement: S1 Table — (DOCX) [file pgph.0003039.s022.docx]

**S1 Table: Additional datasets discussed in the Supplementary Information but excluded from the main text due to data incompleteness and potential biases.**

| **Dataset** | **Location** | **Year(s)** | **Description** | **Sampling method** | **Da­­taset completeness and potential biases** | **Ref.** |
| --- | --- | --- | --- | --- | --- | --- |
| Utah Municipal Wastewater Planning Survey | Utah | 2021 | This survey reported the population receiving collection by each municipal utility that owns or operates a sanitary sewerage system. | The survey completed was by treatment plant operators. Participation was reported in the survey information as being mandatory. | Of those contacted, 71% responded to the survey [60]. In some county subdivisions, >100% of the population appeared connected to sewers, likely due to errors in estimation, differences in how combined treatment and collection facilities reported population estimates, or collection populations residing in neighboring county subdivisions. | [60] |
| Minnesota Wastewater Infrastructure Needs Survey (WINS) | Minnesota | 2021 | Communities (places and townships) responsed to the question "does your community have a collection system?" (yes/no/did not respond). | This was a voluntary survey. | Some communities (number unknown) did not respond to the survey. | [61] |
| Minnesota Subsurface Sewage Treatment Systems (SSTS) | Minnesota | 2017 | This dataset reports the number of subsurface sewage treatment systems (SSTS) in various local government units. | Surveys were sent to local government units with a known SSTS program. According to the SSTS Annual Report [62], ‘some of the data is “hard” data, such as the reported number and types of permits issued. Other data is considered “soft” data, such as the reported best estimates provided to determine SSTS compliance rates and, sometimes, the total number of SSTS in each jurisdiction.’ | Two hundred and eleven out of 218 known SSTS programs submitted an annual report. Not all SSTS programs were identified and contacts were not always provided. Fourteen reported having zero SSTS within their jurisdiction despite permitting SSTS in 2017. | [62] |
