# Supplementary material for: Assessment of sewer connectivity in the United States and its implications for equity in wastewater-based epidemiology
Source: PLOS Glob Public Health. 2024 Apr 17;4(4):e0003039. doi: 10.1371/journal.pgph.0003039 (PMC11023481; doi:10.1371/journal.pgph.0003039)
Supplement: S2 Table — Data are from the 2021 U.S. Census American Community Survey. (DOCX) [file pgph.0003039.s023.docx]

**S2 Table: Counties in the US with >=5% of occupied housing units lacking complete plumbing facilities.** Data are from the 2021 U.S. Census American Community Survey.

|  | **County, State** | **Percent of occupied housing units lacking complete plumbing facilities** |
| --- | --- | --- |
| 0 | Yukon-Koyukuk Census Area, Alaska | 36.1 |
| 1 | Bethel Census Area, Alaska | 31.1 |
| 2 | Kusilvak Census Area, Alaska | 25.3 |
| 3 | Nome Census Area, Alaska | 24.5 |
| 4 | Northwest Arctic Borough, Alaska | 20 |
| 5 | Denali Borough, Alaska | 18.1 |
| 6 | Lake and Peninsula Borough, Alaska | 13.8 |
| 7 | Apache County, Arizona | 12.8 |
| 8 | Dillingham Census Area, Alaska | 12 |
| 9 | Southeast Fairbanks Census Area, Alaska | 11.1 |
| 10 | North Slope Borough, Alaska | 10.8 |
| 11 | Haines Borough, Alaska | 10.5 |
| 12 | Copper River Census Area, Alaska | 10.1 |
| 13 | San Juan County, Utah | 10 |
| 14 | Adams County, Indiana | 9.7 |
| 15 | McKinley County, New Mexico | 9.6 |
| 16 | Oglala Lakota County, South Dakota | 9.2 |
| 17 | Hoonah-Angoon Census Area, Alaska | 7.6 |
| 18 | Mora County, New Mexico | 7.6 |
| 19 | Navajo County, Arizona | 7 |
| 20 | Prince of Wales-Hyder Census Area, Alaska | 6.7 |
| 21 | Lawrence County, Mississippi | 6.2 |
| 22 | Catahoula Parish, Louisiana | 6.1 |
| 23 | Hudspeth County, Texas | 6.1 |
| 24 | Fairbanks North Star Borough, Alaska | 5.6 |
| 25 | Salinas Municipio, Puerto Rico | 5.1 |
| 26 | Yakutat City and Borough, Alaska | 5.1 |
