# Supplementary material for: Assessment of sewer connectivity in the United States and its implications for equity in wastewater-based epidemiology
Source: PLOS Glob Public Health. 2024 Apr 17;4(4):e0003039. doi: 10.1371/journal.pgph.0003039 (PMC11023481; doi:10.1371/journal.pgph.0003039)
Supplement: S3 Table — q-values above 1 are set to 1. (DOCX) [file pgph.0003039.s024.docx]

**S3 Table: Correlation of the percentage of households in a county connected to public sewers with different demographic or economic factors by state; same as Table 3, but including all state-variable pairs.**

| Demographic or economic variable | State | Correlation of variable with % of households by county connected to public sewers (Pearson correlation coefficient) | Significance (q-value) |
| --- | --- | --- | --- |
| Average household size | MN | 0.29 | 0.49 |
|  | IA | -0.24 | 1 |
|  | NJ | 0.34 | 1 |
|  | MD | -0.32 | 1 |
|  | NY | 0.19 | 1 |
|  | FL | -0.12 | 1 |
|  | CA | 0.25 | 1 |
|  | MI | 0.01 | 1 |
| Median age | MN | -0.43 | <0.01 |
|  | MI | -0.41 | 0.01 |
|  | CA | -0.41 | 0.13 |
|  | NY | -0.37 | 0.26 |
|  | MD | 0.17 | 1 |
|  | IA | -0.08 | 1 |
|  | NJ | -0.42 | 1 |
|  | FL | -0.05 | 1 |
| Median income | MN | 0.44 | <0.01 |
|  | FL | 0.48 | <0.01 |
|  | CA | 0.49 | <0.01 |
|  | NJ | -0.27 | 1 |
|  | IA | -0.11 | 1 |
|  | MD | -0.17 | 1 |
|  | MI | 0.21 | 1 |
|  | NY | 0.23 | 1 |
| Percent Hispanic | FL | 0.36 | 0.26 |
|  | NY | 0.31 | 1 |
|  | NJ | 0.51 | 1 |
|  | IA | 0.15 | 1 |
|  | MD | -0.29 | 1 |
|  | MI | 0.24 | 1 |
|  | CA | 0.25 | 1 |
|  | MN | 0.19 | 1 |
| Percent one race and American Indian and Alaska Native | CA | -0.50 | <0.01 |
|  | FL | -0.29 | 1 |
|  | MN | -0.14 | 1 |
|  | NJ | 0.09 | 1 |
|  | NY | 0.03 | 1 |
|  | MI | -0.02 | 1 |
|  | MD | -0.04 | 1 |
|  | IA | -0.01 | 1 |
| Percent one race and Asian | MN | 0.58 | <0.001 |
|  | MI | 0.51 | <0.001 |
|  | NY | 0.55 | <0.001 |
|  | CA | 0.56 | <0.001 |
|  | FL | 0.53 | <0.001 |
|  | IA | 0.22 | 1 |
|  | NJ | 0.25 | 1 |
|  | MD | -0.02 | 1 |
| Percent one race and Black or African American | MN | 0.62 | <0.001 |
|  | MI | 0.59 | <0.001 |
|  | NY | 0.55 | <0.001 |
|  | IA | 0.38 | <0.01 |
|  | CA | 0.46 | 0.02 |
|  | NJ | 0.39 | 1 |
|  | MD | -0.05 | 1 |
|  | FL | 0.01 | 1 |
| Percent one race and Native Hawaiian and other Pacific Islander | IA | 0.26 | 0.85 |
|  | MN | -0.18 | 1 |
|  | MI | -0.15 | 1 |
|  | MD | 0.28 | 1 |
|  | NY | 0.10 | 1 |
|  | CA | 0.07 | 1 |
|  | NJ | -0.07 | 1 |
|  | FL | -0.01 | 1 |
| Percent one race and some other race | NJ | 0.57 | 0.63 |
|  | CA | 0.35 | 0.68 |
|  | MN | 0.26 | 1 |
|  | NY | 0.23 | 1 |
|  | IA | 0.16 | 1 |
|  | MI | 0.17 | 1 |
|  | MD | -0.28 | 1 |
|  | FL | 0.03 | 1 |
| Percent one race and White | MI | -0.57 | <0.001 |
|  | CA | -0.54 | <0.001 |
|  | NY | -0.51 | <0.01 |
|  | IA | -0.35 | 0.03 |
|  | MN | -0.28 | 1 |
|  | NJ | -0.54 | 1 |
|  | FL | -0.08 | 1 |
|  | MD | 0.08 | 1 |
| Percent uninsured (health insurance) | MI | -0.38 | 0.03 |
|  | NJ | 0.51 | 1 |
|  | CA | -0.24 | 1 |
|  | MN | -0.15 | 1 |
|  | NY | -0.10 | 1 |
|  | MD | -0.15 | 1 |
|  | FL | 0.06 | 1 |
|  | IA | -0.03 | 1 |

q-values above 1 are set to 1.
