# Supplementary material for: Assessment of sewer connectivity in the United States and its implications for equity in wastewater-based epidemiology
Source: PLOS Glob Public Health. 2024 Apr 17;4(4):e0003039. doi: 10.1371/journal.pgph.0003039 (PMC11023481; doi:10.1371/journal.pgph.0003039)
Supplement: S4 Table — Only correlations that are significant (q-value< = 0.05) are shown. (DOCX) [file pgph.0003039.s025.docx]

**S4 Table: Correlation of the fraction of households by county connected to public sewers and different demographic or economic factors, stratified by state and Metropolitan, Micropolitan, and Rural Statistical Areas.** Only correlations that are significant (q-value<=0.05) are shown.

| **Demographic or economic variable** | **State** | **Metropolitan, Micropolitan, or Rural Statistical Area** | **Pearson r** | **q-value** |
| --- | --- | --- | --- | --- |
| Median income | FL | Micropolitan Statistical Area | 0.98 | 9.8E-03 |
| Percent Hispanic | FL | Metropolitan Statistical Area | 0.53 | 1.8E-02 |
| Percent one race and Asian | MN | Metropolitan Statistical Area | 0.72 | 2.3E-03 |
|  | FL | Metropolitan Statistical Area | 0.52 | 3.0E-02 |
| Percent one race and Black or African American | MN | Metropolitan Statistical Area | 0.73 | 1.1E-03 |
|  | MI | Metropolitan Statistical Area | 0.67 | 1.4E-02 |
| Percent one race and White | MN | Metropolitan Statistical Area | -0.74 | 7.6E-04 |
|  | MI | Metropolitan Statistical Area | -0.71 | 4.6E-03 |
|  | IA | Metropolitan Statistical Area | -0.69 | 5.0E-02 |
