# Supplementary material for: Assessment of sewer connectivity in the United States and its implications for equity in wastewater-based epidemiology
Source: PLOS Glob Public Health. 2024 Apr 17;4(4):e0003039. doi: 10.1371/journal.pgph.0003039 (PMC11023481; doi:10.1371/journal.pgph.0003039)
Supplement: S5 Table — (DOCX) [file pgph.0003039.s026.docx]

**S5 Table: Manual changes community names reported in the Minnesota Wastewater Infrastructure Needs Survey to be able to match with a U.S. Census Designated Place.**

| **Original name in dataset** | **Edited name for merging with Census Designated Place name** | **Note** |
| --- | --- | --- |
| St X (i.e. St Paul) | St. X (i.e. St. Paul) |  |
| Plainview - Elgin Sanitary District | [Deleted] | There was another entry for Plainview (same zip code) with the same response for whether there was collection facility |
| Clear Lake - Clearwater Sewer Authority | Clear Lake |  |
| Tower-Breitung Wastewater Board | Tower |  |
| Birchwood | Birchwood Village |  |
| Coleraine, Bovey, Taconite Joint Wastewater Commission | [Deleted] | Two entries for Coleraine with same zip code and similar population sizes |
| Glacial Lakes Sanitary Sewer and Water District | [Deleted] | Two entries for Spicer with same zip code and similar population sizes |
| Central Iron Range Sanitary Sewer District | [Deleted] | Two entries for Chisholm with same zip code and similar population sizes |
| Shorewood Park Sanitary District | [Deleted] | Two entries for Rush City with same zip code. Deleted the entry with the smaller population size. |
| Rich Prairie Water & Sewer District (Pierz/Genola) | Pierz |  |
| Pine River Area Sanitary District | Pine River |  |
| Moose Lake Windemere Area Sanitary Sewer District | [Deleted] | Two entries for Moose Lake with same zip code. Deleted the entry with no reported population |
| Alexandria Lakes Area Sanitary District | Alexandria |  |
| North Long Lake Sewer District | Brainerd |  |
| Serpent Lake Sanitary Sewer District | Crosby |  |
| Blomkest/Svea Sewer Board | Blomkest |  |
| GEM Sanitary District | Greenwald |  |
| Chisago Lakes Joint Sewage Treatment Commission | Center City |  |
| Duluth | [Deleted] | 3 entries in Duluth - kept the one with the largest population size (Western Lake Superior Sanitary District (WLSSD)) |
| Duluth – North Shore Sanitary District | [Deleted] | 3 entries in Duluth - kept the one with the largest population size (Western Lake Superior Sanitary District (WLSSD)) |
| MCES - Metro Council Env. Services | [Deleted] | Two entries in St. Paul. Deleted the entry with the smaller population size. |
