# Supplementary material for: Assessment of sewer connectivity in the United States and its implications for equity in wastewater-based epidemiology
Source: PLOS Glob Public Health. 2024 Apr 17;4(4):e0003039. doi: 10.1371/journal.pgph.0003039 (PMC11023481; doi:10.1371/journal.pgph.0003039)
Supplement: Attachment — Submitted filename: Reviewer responses.docx [file pgph.0003039.s027.docx]

Additional Editor Comments (if provided):

(1) The reviewers have made some important suggestions. Please respond to ALL comments by the reviewers.

(2) As pointed by reviewer 1, the rationale for a deep-dive using the Florida Department of Health onsite sewage treatment and disposal systems inspection data is unclear. Please provide a clear justification of the utility of this analysis for this paper.

We appreciate the editor and reviewer 1’s comments and the opportunity to improve the clarity of this section. We did not intend to do a deep-dive specifically into Florida only. Rather, in the absence of comprehensive national data, this was one of multiple datasets that we identified to address the question of which geographic areas have low connectivity to sewers. To clarify our selection of datasets, we added the following text to the results in lines 295-298:

*In the absence of comprehensive national data on sewer connectivity, we assembled publicly available state-based datasets, which included California, Florida, Iowa, Maryland, Michigan, Minnesota, New Jersey, and New York, as well as the U.S. Island Areas.*

(3) Please use the most recent datasets, wherever possible. If not, please discuss the potential limitations of using data that are more than a decade old (for example, the data from EPA Clean Watersheds Needs Survey is from 2012).

We have used the most recent datasets available, and in fact one of our findings is the need for more comprehensive and updated datasets to address the question of sewer connectivity (see the Data limitations section in Discussion from lines 496-507). We note, for example, that the most recent EPA Clean Watersheds Needs Survey was from 2012. In response to the editor’s point, we have added the following text in this section (lines 505-507) to highlight the importance of updated datasets:

*Additionally, multiple datasets were over a decade old, and changes to which households are served by sewer and septic systems may have occurred since the datasets were collected.*

(4) Please provide more details about the justification of the model selection (SIR models vs other compartmental/meta-population models) and the assumptions for base parameter values used in the simulation models for exploring the applicability of WBE data to neighboring un-sampled communities as the findings have wider implications for wastewater-based surveillance.

Our goal was to explore the impact of factors on the generalizability of wastewater rather than to accurately capture the dynamics in all scenarios; as such, we chose a simple susceptible-infected-recovered compartmental model (SIR). This has been updated in the Discussion lines 538-540:

*As the purpose of our model was to explore the impact of factors on the generalizability of wastewater rather than to accurately capture the dynamics in all scenarios, we used a compartmental susceptible-infected-recovered model (SIR) and made simplifying assumptions…*

The base parameters were chosen to be roughly similar to SARS-CoV-2. We varied the parameters in the sensitivity analyses (see response to Reviewer 1 below) and found that our main results were unaffected.

Reviewer #1: This paper provides an overview of the potential for WBE based on sewered connections in the United States. However, non-sewered sanitation systems can, and are also sampled and excluded from this analysis.

Thank you for raising this point. We agree that technology for sampling non-sewered sanitation systems is being developed and has been applied in some settings. However, here we focused on understanding connections to sewer systems because wastewater sampling in the U.S. is currently more commonly done on sewered systems (i.e., CDC NWSS and WastewaterSCAN) and there aren’t large publicly-available datasets of non-sewered settings. We discuss non-sewered sanitation systems in the Discussion under “Additional considerations for equity in WBE”, and we added the following references:

- Pocock et al., Proof of Concept Study: Application of wastewater-based surveillance to monitor SARS-CoV-2 prevalence in South African communities, Report to the Water Research Commission, 2020, <https://wrcwebsite.azurewebsites.net/wp-content/uploads/mdocs/TT%20832-20%20final%20web.pdf>
- Barnes et al., Utilizing river and wastewater as a SARS-CoV-2 surveillance tool in settings with limited formal sewage systems, Nature Communication, 2023, <https://www.nature.com/articles/s41467-023-43047-y>

Also missing is a comparison to how many sites are currently sampling for WBE compared to this research on the potential application.

We appreciate the opportunity to clarify the main goal of our manuscript. While we recognize that it would be interesting to characterize which sites are sampling for WBE, we aim to address a set of questions that center on to what extent there is demographic and economic inequity in sewer connectivity. Nonetheless, to the reviewer’s point, we have added the following references to the text on the importance of considering equity when choosing sites for sampling in the Discussion section:

- Hu et al., Assessing health equity in wastewater monitoring programs: Differences in the demographics and social vulnerability of sewered and unsewered populations across North Carolina, medRxiv, 2023, <https://www.medrxiv.org/content/10.1101/2023.10.06.23296680v1>
- Naughton et al., Show us the data: global COVID-19 wastewater monitoring efforts, equity, and gaps, FEMS Microbes, 2023, <https://academic.oup.com/femsmicrobes/article/doi/10.1093/femsmc/xtad003/6986253>

1. Introduction is missing several key references and literature on this subject. At a minimum, suggest considered 3 additional references:
a. Burden of disease attributable to unsafe drinking water, sanitation, and hygiene in domestic settings: a global analysis for selected adverse health outcomes
b. The effects of racism, social exclusion, and discrimination on achieving universal safe water and sanitation in high-income countries
c. Using wastewater to overcome health disparities among rural residents

Thank you for suggesting these references; we have added them. We found the following additional references that provide context on these subjects, and we have added them to the introduction.

- Rural Community Assistance Partnership, Still Living without the Basics in the 21st Century: Analyzing the Availability of Water and Sanitation Services in the United States, 2017, <https://www.rcap.org/wp-content/uploads/2017/05/Still-Living-Without-the-Basics-Water.pdf>

# Leker et al., PLOS One, 2018, Relationship between race and community water and sewer service in North Carolina, USA <https://journals.plos.org/plosone/article?id=10.1371/journal.pone.0193225>

# Meehan et al., Geographies of insecure water access and the housing–water nexus in US cities, PNAS, 2020, <https://www.pnas.org/doi/10.1073/pnas.2007361117>

2. Methods, why was a convenience sample done and not the entire US as the title states? What were the criteria for this convenience. Why was Florida chosen as the dataset up to 2012 which is more than 10 years old.

We would have preferred comprehensive national data to catalog the extent to which there is socioeconomic and demographic variation in access to sewer systems. However, such data are not available–an important point that we raise in this manuscript. In the absence of publicly available comprehensive US national data, we sought to address our questions through assembling a set of state-based datasets informed (1) by our discussions with experts in the federal Environmental Protection Agency and in multiple states’ agencies and (2) by web searches for relevant datasets.

At the state-level, we analyzed data from the EPA on collection facility locations and populations served in 8 states (see main text lines 342-356, Tables 3, S2, and S3, and Figure S11). Additionally, we analyzed datasets from the 4 island areas of Guam, the Northern Mariana Islands, the Virgin Islands, and American Samoa, discussed in the main text (lines 363-365) and elaborated on them in the S2 Appendix. We also analyzed datasets from Utah and Minnesota (Figures S13, S15, and S16). However, these latter datasets were incomplete and biased, in that they had limited and uneven coverage of the population and the reported sewer locations, and we ran into the challenge that sewershed boundaries do not always overlap with administrative boundaries. Due to these biases and space limitations in the main text, we opted to include them as Supplemental Material. The Florida septic tank dataset was comprehensive and offered high spatial resolution, though as the reviewer noted it dated to 2012 (Table 1). We did not find a more recent version of this dataset.

While other datasets exist–for example, datasets on septic tank locations in Hawaii, Delaware, and Rhode Island (EPA, 2021) and data on public sewer systems in North Carolina (NC OneMap)--it was not clear that these would extend our findings or result in generalizable conclusions because they were subject to individual biases (e.g., incomplete or biased reporting).

We have updated the description on how we compiled the data in the Methods section lines 92-96 to:

*We assembled publicly available datasets on sewer connectivity, septic connectivity, or no plumbing at the household level across the U.S. from federal and state agencies informed by (1) our discussions with experts from federal and state agencies and (2) web searches using terms including “Number of people connected with treatment plants in United States”, “Number of households on septic”, and “U.S. census sewer”.*

We have also updated the motivation for the Results section on connectivity to sewers by geographic area in lines 295-298 to:

*In the absence of comprehensive national data on sewer connectivity, we assembled publicly available state-based datasets, which included California, Florida, Iowa, Maryland, Michigan, Minnesota, New Jersey, and New York, as well as the U.S. Island Areas.*

References:

U.S. Environmental Protection Agency, Office of Water. Report to Congress on The Prevalence Throughout the U.S. of Low- and Moderate-Income Households Without Access to a Treatment Works and The Use by States of Assistance under Section 603(c)(12) of the Federal Water Pollution Control Act. U.S. Environmental Protection Agency, 2021, <https://www.epa.gov/system/files/documents/2022-01/low-mod-income-without-treatment_report-to-congress.pdf>

NC OneMap, <https://www.nconemap.gov/datasets/nconemap::type-a-current-public-sewer-systems-2004/about>

3. I am surprised by the rural and urban dataset that is so old. WHO and UNICEF have new data out on the United States:
a. World Health Organization (WHO) and the United Nations Children’s Fund (UNICEF). Progress on household drinking water, sanitation and hygiene 2000-2022. 2023. <https://data.unicef.org/resources/jmp-report-2023/>

We thank the reviewer for pointing out the country-level WHO and UNICEF dataset. The country-level data for the United States is from the American Housing Survey, which is the same data that we used. However, we and others have found heterogeneities within countries that would be obscured when aggregated to the country-level, limiting our ability to address our main questions. Consequently, we focused on higher resolution state-level data.

The American Housing Survey (AHS) 2015 and later public use files (PUF, publicly-available data by household) do not include many of the geographic indicators included in prior years’ PUFs due to disclosure avoidance procedures required by the Census Bureau that were updated with the update to the AHS in 2015 (Getting Started with the Public Use File: 2015 and Beyond, 2022, <https://www.census.gov/programs-surveys/ahs/tech-documentation/help-guides/2015-later/puf_start.html>). Thus, the most recent data from the AHS that includes information about urban and rural location of the household is from 2013 (since the survey is administered every other year).

We did find the reference from WHO and UNICEF that the reviewer suggested to be helpful in thinking about international sewer connectivity and have added the reference into the Discussion.

4. Section “Is there inequity in who is sampled by wastewater?” is not accurate to the data presented. This section is instead the portion of the population that could be sampled by WBE.

We thank the reviewer for this point. The section header has been changed to “Is there inequity in who could be sampled by wastewater-based epidemiology?”.

5. Have you considered also the role of CSO and SSO in Figure 1? This is important for dilution of samples.

We agree that whether sewage goes into a CSO and SSO system could impact the dilution of samples and the interpretation of the wastewater data. However, wastewater processing was not at issue in our study. Instead, we focused on connectivity. Figure 1 shows how people responded to the question of whether their home is connected to a sewer system, and they were not asked to specify what kind of sewer system.

6. The portion on sewer connectivity by racial group is missing important context for infrastructure inequalities. Without that, this section is not in context.

We added the following text in the introduction (lines 56-58) about how disparities in sewer connectivity are driven by structural inequalities.

*Variability in sewer connectivity exists across the U.S. [14,15], with more connectivity in urban areas than rural areas [11,16,17] and disparities driven by structural inequalities [18].*

7. The section on Florida should be removed or add additional states. Otherwise, what is the context or rationale for a single state deep dive?

We analyzed 8 states in addition to Florida using the EPA data, and this is described in the same section of the results as the Florida data. We included analyses of Utah and Minnesota in the Supplementary Information, noting that those datasets have limited and uneven coverage, and sewershed boundaries don’t always overlap with administrative boundaries, both of which limit interpretability.

8. Table 2 Significance, round to 2 significant figures or state <.05. E-07 is too many significant figures.

We updated the q-values in Table 2 to state <0.001, <0.01, or the value. We chose these options in order to give more of a dynamic range of significance values.

9. Table 3 Significance, round to 2 significant figures or state <.05. Table 3 would be better to have all states.

We updated the q-values for Table 3 as per Table 2 in the previous comment. Including all correlations for the 8 states would result in 56 rows (7 variables * 8 states). As such, we included only the state-variable combinations that had a statistically significant correlation in the main text table and put the full set in the Supplemental Information at Table S2.

10. In the section “Factors influencing applicability of wastewater-based epidemiology to communities lacking sewer connection” a lot more citations are needed to ground these assumptions. Reproduction number of 1.5 in particular. What other SIR models have been conducted (there have been many the authors are missing).

We appreciate the opportunity to clarify that the goal of the model was to evaluate the extent to which wastewater-based epidemiological results extend to nearby non-sewered or less-sewered communities, rather than to address what would happen in a specific disease scenario. As such, our goal was not to choose specific parameters, but rather to assess which factors influence the reliability of generalizing WBE from a well-sampled community to these non- or less-sewered communities. Thus, we varied the reproduction number (*R*_0_=1.5-3) and the recovery rate (Ɣ_I_=0.1-0.33) (see lines 423-426 and Figure S12). We found that, in keeping with intuition, as *R*_0_ increases, the time difference between the peak in wastewater and in population B also decreases. Varying the recovery rate resulted in only a small effect on peak timing.

We are not aware of other papers that have tackled this question. Compartmental models have been previously used to analyze wastewater data, which we have now cited an example of in Discussion of the manuscript, but the goals of these studies were to estimate the disease dynamics rather than to evaluate the impact of sewer connectivity on the generalizability of wastewater data as we have done here. We would welcome the reviewer’s suggestions for any additional papers we should cite.

11. Compare the results of this work to how many locations are currently being sampled by BioBot or CDC NWSS for WBE.

While we agree with the reviewer that it would be interesting to evaluate the equity of specific sampling schemes, our goal is to investigate the heterogeneity in the demographic, socioeconomic, and geographic characteristics of populations with sewer access in the US. We wish to clarify that our analyses do not and are not intended to specify a sampling strategy.

There are studies defining the demographics of those sampled in a variety of different contexts (see above in the response to “Also missing is a comparison to how many sites are currently sampling for WBE compared to this research on the potential application.”); we cited these in the Discussion.

Reviewer #2: The manuscript by Yu et al. addresses a timely and significant question regarding the spatial coverage of municipal based wastewater surveillance for infectious disease (although clearly their data and analysis could be applied for detection of non-microbial analytes, e.g. opioids, in wastewater). Although it is well-documented that there is a significant percentage of the U.S. population that is not connected to municipal wastewater, the “who” and “where” of this population is not well understood. The authors use a diverse set of data sources to better understand this population. This is important as sewersheds do not align with political or demographic boundaries and are determined by a diversity of factors, including topography.

Their descriptive data reproduces prior studies indicating that the assumption that the unsewered population would be uniformly more or less wealthy is incorrect—there is substantial diversity based on income, region, and locality, which reflects, at least in part, ethnicity. The use of multiple data sets and appropriate statistical analysis is an important advance on current knowledge. The data clearly show that first nation’s populations living in Alaska and the Navajo Nation are significantly overrepresented in being unconnected to municipal systems.

The modeling aspect of the study addresses a critical question in optimal coverage of a national surveillance system—“is the unsewered and thus unmonitored population informed by proximity and contact with a sewered and monitored population”? Although there is certainly room to expand this model to different levels of contact, including “mass participation events” such as sporting events or seasonal shifts in contact, the presented model is a valuable addition and lays the groundwork for future modeling and comparison to epidemiological data.

Thank you so much for the encouraging feedback on our work!

Reviewer #3: Overall, I believe this to be a broad analysis of factors contributing to potential inequities in the implementation of wastewater based epidemiology, and presents a summary of potential biases which would be reflected in a wastewater signal. My only major comment is that median income should be analyzed as a factor, particularly when some of the factors analyzed are likely to be nearly perfectly collinear.

Thank you for this comment. We have now analyzed household income (see below).

Line 65-68: Wealthier with respect to local rural or urban area or wealthier compared to national statistics? Wording isn't great.

We have updated the text (lines 69-72) to the following to try to clarify what we meant, but please let us know if it is still not clear.

*Based on the 2019 U.S. Census Bureau’s American Housing Survey, rural areas are less connected to sewers, and while the income in rural areas was lower than that in metropolitan areas, households connected to septic tanks were wealthier than those connected to sewers within both rural and metropolitan areas.*

Line 125-127: Does sub-sampling for areas with a minimum number of households and population not bias your average sewered % up by omitting some potentially very rural areas? I would appreciate a % of total data included in the study.

We checked the % of total data included in the study, and it was >99% of the population and >99.7% of the number of households for all analyses of geographic distribution of sewer connectivity. Because of the high number of households included, we do not expect that omitting the counties or county subdivisions with less than 20 population size or 5 households would bias the results. We have added this information to the methods section as the following text (lines 144-145):

*This thresholding removed at most 1% of the total population and at most 0.03% of the total number of households, making it unlikely to bias our results.*

Methods general: The number of observations used for your results should be included somewhere.

We have included the number of observations for Figures 1, 2, and 3 in Figures S2 and S3. We put the information on the number of observations into the SI in an effort to limit clutter in the main text figures. We have also included the number of observations for Figure S1. We didn’t know how to add the number of observations for Figure S4 without it looking too messy, but we have indicated in the legend to Figure S4 that the number of observations can be found in the code repository.

Results general: Some of the results suffer from the confounding effect of wealth. For example, householder age and the proposed correlation are likely to reflect average income and poverty, with age simply being colinear. Household income is part of the AHS, and should have been analyzed rather than the binary in poverty/not in poverty.

We thank the reviewer for this point. We have updated our analyses to include household income (defined as a percentage of the federal poverty threshold) (Figures 2, 3, S4). We see decreasing sewer connectivity by increasing income except for a few exceptions in the Mountain census division and certain metropolitan areas (similar exceptions as we saw before when we were looking at whether households are in poverty or not in poverty). We have updated the main text to (lines 230-234):

*Connection to sewers decreased with household income, as measured as a percentage of the federal poverty threshold determined by the Census [37], with the exception of the Mountain region where households with an income at 50-99% of the poverty level were less connected than those with income at ≥200% of the poverty level (84.5% vs 88.2% of households).*

We also calculated the correlation between householder age and household income in the American Housing Survey dataset. We find a slight negative correlation between the two variables in all census divisions. Thus, the decrease in sewer connectivity by age and the decrease in sewer connectivity by income cannot be explained by collinearity between age and income; they would need to be positively rather than negatively correlated to explain these trends. We have added the following text to the discussion (lines 453-455) and Figure S13 to address this point.

*The decrease in sewer connectivity by income could not be explained by collinearity between age and income as age and income exhibited a weak negative correlation (Fig S13).*

Fig S 13: Correlation between householder age and household income in the American Housing Survey dataset by census division.

We have also added the following text to the discussion (lines 465-467) about how we have not attempted to determine which variables drive the differences in sewer connectivity and how some of the variables are likely to be correlated with one another:

*We have not attempted to determine why the observed differences in sewer connectivity exist. Many of the variables that we analyzed are likely to be correlated with one another.*

Additional changes:

We noticed a typo in the equations for the SIR model on pgs. 55-56 of the manuscript and have corrected them. Previously, the equations for dI_A_/dt and dI_B_/dt contained the parameters β_AA_, β_AB_, β_BA_, and β_BB_, but these should have been β_A_, β_A_, β_B_, and β_B_, respectively. The typos were only in the manuscript and did not affect the model itself.
